# Acid-base homeostasis orchestrated by NHE1 defines the pancreatic stellate cell phenotype in pancreatic cancer

**DOI:** 10.1172/jci.insight.170928

**Published:** 2023-10-09

**Authors:** Zoltán Pethő, Karolina Najder, Stephanie Beel, Benedikt Fels, Ilka Neumann, Sandra Schimmelpfennig, Sarah Sargin, Maria Wolters, Klavs Grantins, Eva Wardelmann, Miso Mitkovski, Andrea Oeckinghaus, Albrecht Schwab

**Affiliations:** 1 Institute of Physiology II and; 2 Institute of Molecular Tumor Biology, University of Münster, Münster, Germany.; 3 Institute of Physiology, University of Lübeck, Lübeck, Germany.; 4 Gerhard-Domagk-Institute of Pathology, University of Münster, Münster, Germany.; 5 City Campus Light Microscopy Facility, Max Planck Institute for Multidisciplinary Sciences, Goettingen, Germany.

**Keywords:** Metabolism, Oncology, Epithelial transport of ions and water, Fibrosis, Homeostasis

## Abstract

Pancreatic ductal adenocarcinoma (PDAC) progresses in an organ with a unique pH landscape, where the stroma acidifies after each meal. We hypothesized that disrupting this pH landscape during PDAC progression triggers pancreatic stellate cells (PSCs) and cancer-associated fibroblasts (CAFs) to induce PDAC fibrosis. We revealed that alkaline environmental pH was sufficient to induce PSC differentiation to a myofibroblastic phenotype. We then mechanistically dissected this finding, focusing on the involvement of the Na^+^/H^+^ exchanger NHE1. Perturbing cellular pH homeostasis by inhibiting NHE1 with cariporide partially altered the myofibroblastic PSC phenotype. To show the relevance of this finding in vivo, we targeted NHE1 in murine PDAC (KPfC). Indeed, tumor fibrosis decreased when mice received the NHE1-inhibitor cariporide in addition to gemcitabine treatment. Moreover, the tumor immune infiltrate shifted from granulocyte rich to more lymphocytic. Taken together, our study provides mechanistic evidence on how the pancreatic pH landscape shapes pancreatic cancer through tuning PSC differentiation.

## Graphical abstract

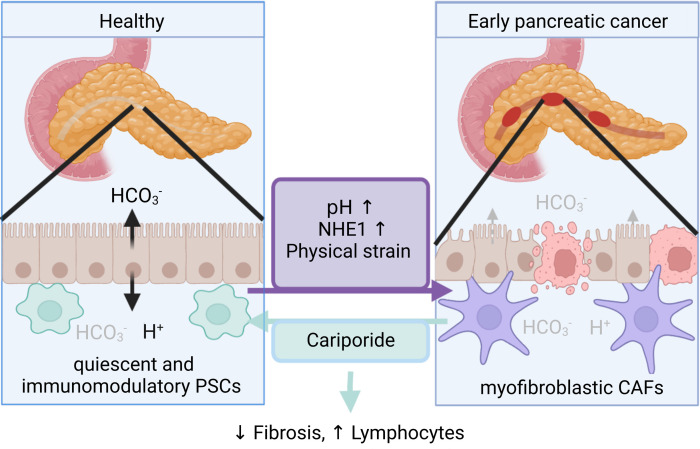

## Introduction

Pancreatic ductal adenocarcinoma (PDAC) has a detrimental prognosis, with an overall 5-year survival rate of 12% even with state-of-the-art therapy (1). The main limiting factors of PDAC therapy are early metastases and ineffective radio- and chemotherapy. The latter is caused, partially, by the predominant presence of stromal cells and extracellular components within the tumor mass (2). The stiff fibrotic extracellular matrix (ECM) of the tumor stroma, produced mainly by pancreatic stellate cells (PSCs), limits the diffusion of nutrients, rendering the PDAC stroma hypoxic and acidic compared with the healthy pancreas (3). Moreover, fibrosis hampers the antitumor immune response, as the cytotoxic CD8^+^ lymphocytes are substituted by neutrophil granulocytes and myeloid-derived suppressor cells (MDSCs) (reviewed in refs. 4, 5).

In the healthy pancreas, PSCs are localized near acinar, and ductal cells and are in a so-called quiescent state (6). In PDAC, PSCs are a heterogeneous cell group mainly functioning as protumor cancer-associated fibroblasts (CAFs) (7). On the one hand, PSCs differentiate into myofibroblastic CAFs (myCAFs) upon noxious stimuli such as inflammation, hypoxia, or mechanical stress. They express activation markers such as α-smooth muscle actin (αSMA), migrate to the site of injury, proliferate, and remodel the ECM. On the other hand, PSCs can differentiate into immunomodulatory CAFs (iCAFs), attracting a wide range of tumor-promoting and antitumor immune cells. However, it is important to note that CAFs are a heterogeneous group of cells derived from different lineages and do not only originate from PSCs. Multiple therapeutic strategies — e.g., cellular reprogramming with vitamin D — target tumor-promoting CAFs in pancreatic cancer (8, 9). However, none of these therapies exploits the unique pancreatic pH landscape, detailed below.

Acid-base homeostasis is a fundamental physiological function of every cell. The intracellular pH (pH_i_) is usually slightly more acidic (ΔpH_i_ = 0.2) than the extracellular pH (pH_e_) (10). Numerous pH-sensory and pH-regulatory molecules maintain pH_i_. Perturbed pH_e_ can be directly sensed by GPCRs and other pH-sensing membrane proteins — e.g., ion channels (11). Cells use acid extruders — such as the Na^+^/H^+^ exchanger NHE1 (*Slc9a1*), the Na^+^/HCO_3_^–^ cotransporter NBC1 (*Slc4a4*), or the V-type H^+^-ATPase (*Atp6v0*) — in case of intracellular acidification (10). From these transporters, NHE1 is particularly well druggable; multiple validated inhibitors are available, from which cariporide has also been tested in large clinical trials (12, 13). In cancer cells, pH homeostasis is consistently dysregulated. Their pH_i_ is usually higher than pH_e_ (14) because of increased expression of pH-regulating transporters such as NHE1 (15–18). However, how pH regulation is altered in PSCs during PDAC is unknown.

The healthy pancreas has a unique pH landscape (19). Upon each meal, ductal cells secrete up to ~150 mM HCO_3_^–^ into the ductal lumen (20). This, in turn, leads to an equimolar efflux of H^+^ across the basolateral membrane into the stroma. Otherwise, the ductal epithelial cells would be unable to maintain their pH_i_ homeostasis. Consequently, all cells residing in the pancreatic stroma, such as PSCs, are intermittently exposed to a substantial acid load, detected in proof-of-principle measurements with pH microelectrode (21). However, in our view, the reported values are an underestimation of the postprandial interstitial acidosis of the pancreas. How PSCs react to such an environmental acidosis has not been investigated to date. It is plausible that tumorous transformation of the ductal cells in the early stages of pancreatic tumorigenesis (in pancreatic intraepithelial neoplasia [PanIN]) impairs ductal cell polarity and, thus, apical HCO_3_^–^ secretion into the ductal lumen (19). Less apical HCO_3_^–^ secretion results in reduced H^+^ extrusion into the pancreatic stroma and, hence, in attenuated acidification or relative alkalinization of the interstitial pH. We hypothesize that this prolonged decrease in intermittent acidity is a major determinant of the PSC phenotype and leads to tissue fibrosis in PDAC. We tested this hypothesis by combining in vitro and in vivo experiments focusing on pH regulation and PSC and CAF phenotypes, with the aim of better understanding desmoplasia in PDAC and identifying a potential therapeutic target for its disruption.

## Results

### Altered environmental pH triggers a PSC phenotype switch in vitro.

**Figure 1:**
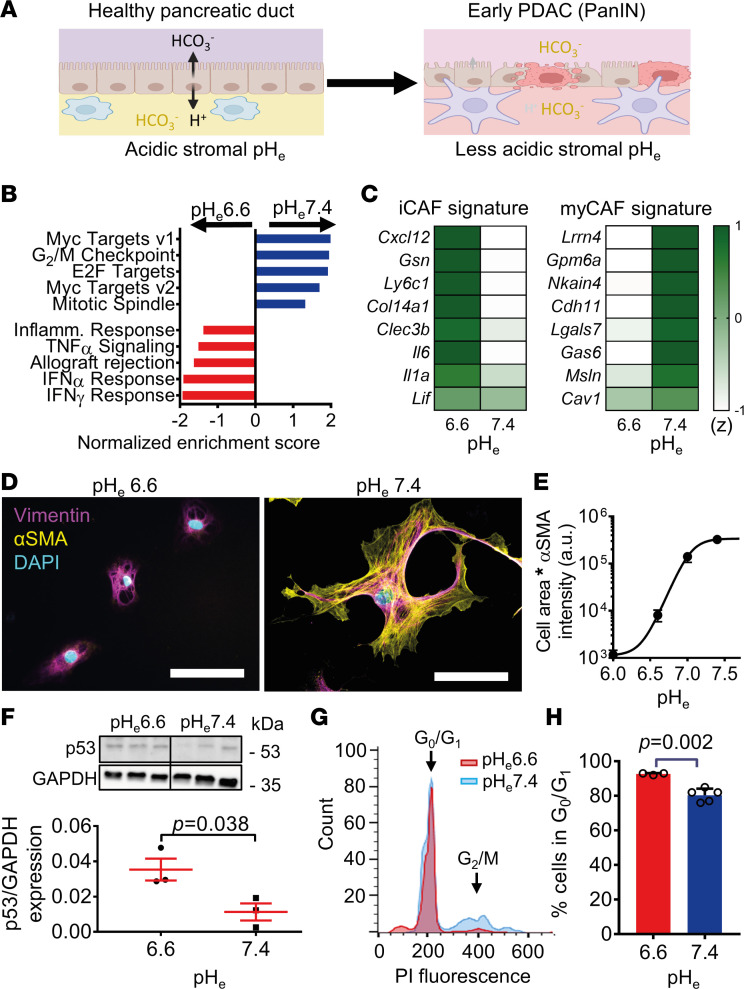
Environmental alkalization induces myofibroblastic PSC differentiation and proliferation. (**A**) Concept of the working hypothesis. In the healthy pancreas, the marked HCO_3_^–^ secretion upon each meal results in a distinct stromal acidification, keeping the PSCs in a quiescent nonfibrotic phenotype. Upon malignant transformation in early PDAC (PanIN), ductal secretion decreases, resulting in a relief of the intermittent acidity ( = relative stromal alkalization), leading to a myofibroblastic PSC differentiation. Acidic → alkaline pH_e_ is depicted by yellow → purple colors. (**B**) Hallmark gene set enrichment analysis (GSEA) of RNA-Seq data from PSCs cultured at pH_e_7.4 versus pH_e_6.6 shows the top 5 differentially regulated pathways (*n*/*N* = 3/3). (**C**) A heatmap of RNA-Seq expression mean *Z* scores computed for published signature genes of immunomodulatory CAFs (left) and myofibroblastic CAFs (right). The gene (rows) *Z* scores for pH_e_6.6 and pH_e_7.4 are color coded. Dark green indicates higher expression *Z* scores (*n*/*N* = 3/3). (**D**) Immunocytochemistry images of PSCs cultured at pH_e_6.6 or pH_e_7.4. The activation marker αSMA (yellow) and the general PSC marker vimentin (magenta) are labeled. Nuclei are stained with DAPI (cyan). Scale bar: 50 μm. (**E**) Cell areas multiplied by αSMA intensity is taken as a readout for the myofibroblastic PSC phenotype. It is plotted as a function of pH_e_. Mean values are shown as *n* ≥ 142 from *N* = 3 mice. Half-maximum (EC_50_) pH_e_-dependent PSC activation occurs at pH_e_7.0. Note the logarithmic scale of the ordinate. (**F**) Representative Western blot (top) of p53 and GAPDH of PSCs cultured at pH_e_6.6 or pH_e_7.4, with subsequent quantification (bottom) (*n*/*N* = 3/3). (**G**) Representative cell cycle histogram of PSCs cultured at pH_e_6.6 (red) and pH_e_7.4 (blue), assessed by flow cytometry with propidium iodide (PI) staining. Cell populations at different stages of the cell cycle are indicated by arrows. (**H**) The bar chart depicts the percentage of PSCs at the G_0_/G_1_ phase of the cell cycle when cultured at pH_e_ 6.6 (red) and pH_e_7.4 (blue). Data points are *n*_pHe6.6_ = 3 and *n*_pHe7.4_ = 5 measurements from *n* = 3 individual mice. Statistical tests in **F** and **H** were performed with 2-tailed unpaired Student’s *t* tests. **A** was created with BioRender.com.

First, we aimed to illustrate the impact of environmental pH on the PSC phenotype in the healthy and cancerous pancreas (Figure 1A). We performed an unbiased screening using RNA-Seq of primary murine PSCs cultured at pH_e_6.6 and pH_e_7.4 (GSE223205; https://www.ncbi.nlm.nih.gov/geo/query/acc.cgi?acc=GSE223205). This pH range can plausibly occur locally in the pancreas stroma and along the course of PDAC (3). The replicates in each group were homogeneous according to principal component analysis (Supplemental Figure 1A; supplemental material available online with this article; https://doi.org/10.1172/jci.insight.170928DS1). We found a total of 1,769 genes differentially regulated between the 2 groups (log_2_FC > 0.58, *P* < 0.05), with 804 genes being upregulated at pH_e_7.4 and 965 genes being upregulated at pH_e_6.6 (Supplemental Figure 1B and Supplemental Table 1). A gene set enrichment analysis (GSEA) (Figure 1B and Supplemental Figure 1, C and D) revealed that cell proliferation, communication, adhesion, and cell cycle pathways are markedly upregulated in PSCs cultured at pH_e_7.4. In contrast, immune response–related pathways are upregulated at pH_e_6.6. Next, we compared our results with gene expression signatures of published PSC-derived CAF subpopulations (7, 22) (Figure 1C). Genes from iCAFs were highly expressed at pH_e_6.6, and PSCs cultured at pH_e_7.4 highly expressed myCAF marker genes (Supplemental Table 3). These findings suggest that PSCs isolated from the healthy pancreas with a physiological history of intermittent acidification have an immunomodulatory phenotype when kept in an acidic environment. However, they acquire a myofibroblastic phenotype upon removal of the extracellular acidity when kept at pH_e_ 7.0 and 7.4. Therefore, we will designate them as iPSCs and myPSCs, respectively.

To confirm the pH_e_-dependent phenotype switch of PSCs, we cultured freshly isolated PSCs from WT mice in media with a pH range from pH_e_6.0 to pH_e_7.4 for 72 hours and then quantified αSMA expression as a derivative of the myofibroblastic phenotype. For a more robust readout of myofibroblastic PSC phenotype and αSMA quantity, we multiplied cell area with αSMA intensity. As expected from the RNA-Seq data, the myCAF phenotype heavily relied on the environmental pH (Figure 1, D and E). The highest expression of αSMA was found at pH_e_7.4 (pH_e_6.0: 1,166 ± 146 a.u., pH_e_6.6: 8,090 ± 1,137 a.u., pH_e_7.0: 140,365 ± 17,508 a.u., pH_e_7.4: 323,756 ± 30,015 a.u.; *n* ≥ 142 cells from *N* = 3 mice). Western blot experiments comparing αSMA normalized to vimentin protein levels in PSCs cultured at pH_e_6.6 versus pH_e_7.4 recapitulated the immunocytochemistry data (αSMA/vimentin protein expression) (pH_e_6.6: 0.11 ± 0.08 a.u., pH_e_7.4: 1.23 ± 0.18 a.u., *P* = 0.015, *n* = 4 samples from *n* = 4 mice) (Supplemental Figure 2, A and B). The percentage of viable cells in the population was reduced at pH_e_6.0 (12% ± 2%) and pH_e_6.3 (31% ± 5%), while it was hardly affected at pH_e_ > 6.5 (pH_e_6.6: 83% ± 2%; pH_e_7.0: 96% ± 1%; pH_e_7.4: 94% ± 2%, cells from *n* = 5 fields of view of *n* = 5 mice, *P* < 0.0001) (Supplemental Figure 2, C and D). Increased cell proliferation of myPSCs was underlined by the fact that p53 protein was more expressed at pH_e_6.6 than at pH_e_7.4 (Figure 1F) (relative p53 expression to GAPDH: pH_e_6.6: 0,04 ± 0.01, pH_e_7.4: 0.01 ± 0.01;lysates from *N* = 3 mice each, *P* = 0.038). Cell cycle progression was also diminished at pH_e_6.6 compared with pH_e_7.4 (Figure 1, G and H; percentage of PSCs in G_0_/G_1_ after 72 hours: pH_e_6.6: 93 ± 1%; pH_e_7.4: 80 ± 2%; *n* ≥ 3 measurements from *N* = 3 mice, *P* = 0.017). Taken together, environmental pH was crucial in tuning the differentiation of PSCs. They acquired iPSC and myPSC phenotypes at pH_e_6.6 and pH_e_7.4, respectively.

### NHE1 is a key regulator of PSC pH homeostasis.

**Figure 2:**
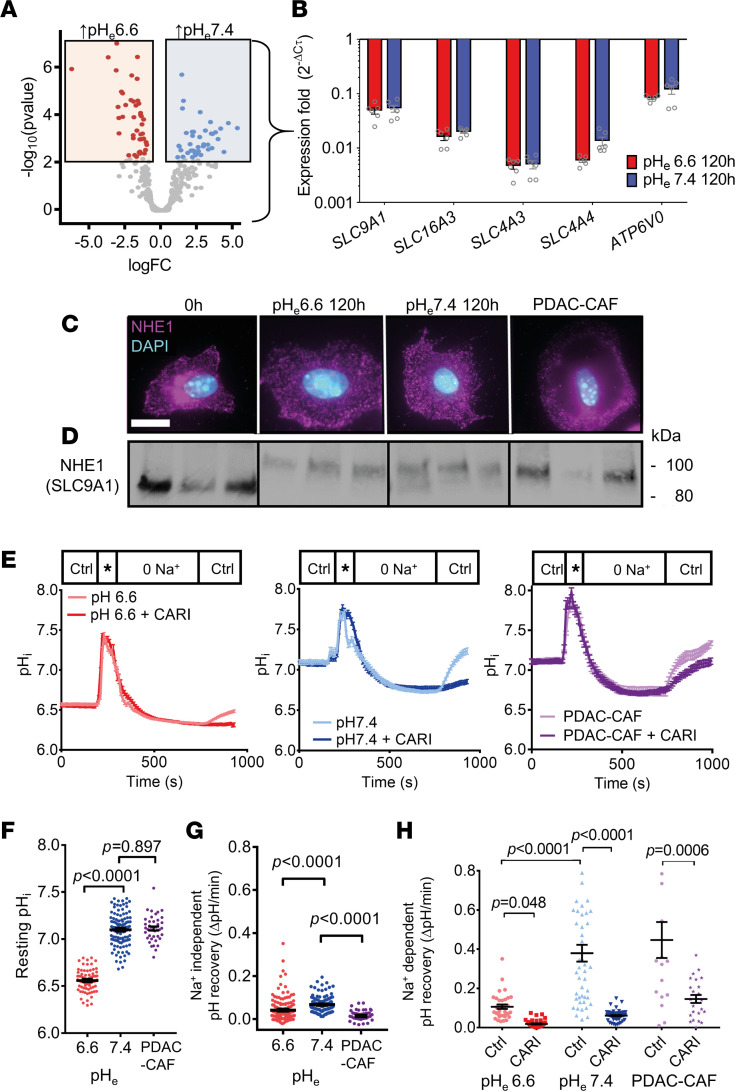
NHE1-mediated pH recovery is inhibited by cariporide in PSCs. (**A**) Volcano plot analysis of ion transporter genes (GO:0015075) derived from the RNA-Seq data of PSCs cultured at pH_e_7.4 and pH_e_6.6 (*n*/*N* = 3/3). Genes indicated by red (*n* = 43) and blue (*n* = 44) dots highlighted in rectangles are upregulated at pH_e_6.6 and pH_e_7.4, respectively. (**B**) Subsequent qPCR validation of ion transporter gene expression levels by the 2^–ΔCT^ method compared with the housekeeping genes *Ywhaz* and *18s* rRNA. Bar charts show mean expression of genes from freshly isolated quiescent PSCs (0 hours, white) and PSCs cultured at pH_e_7.4 (blue) or pH_e_6.6 (red) (*n*/*N* = 6/3). (**C**) Representative immunofluorescence images showing the cellular localization of NHE1 in freshly isolated quiescent PSCs (0 hours), PSCs cultured at pH_e_7.4 or pH_e_6.6 for 120 hours, or vehicle-treated KPfC-derived PSCs (PDAC-PSC) (NHE1: magenta; DAPI: blue). Scale bar: 10 μm. (**D**) NHE1 Western blots are shown, with the top bands at 100 kDa corresponding to the glycosylated NHE1, whereas bands with lower molecular weight (80 kDa) correspond to the unglycosylated NHE1. Lysates are from *N* = 3 mice each. (**E**) pH_i_ recordings of WT PSCs cultured at pH_e_6.6 (left) and pH_e_7.4 (middle) or KPfC-derived CAFs (cultured at pH_e_7.4; right). pH_i_ was acidified temporarily by applying the NH_4_^+^ prepulse (*) technique. NHE1-independent pH_i_ recovery starts when pH_i_ has reached its minimum in the presence of the Na^+^-free solution (“0 Na^+^”). NHE1-dependent pH_i_ recovery can be observed in the last step (Ctrl) of the experiment when cariporide was added to the Na^+^-containing superfusion as indicated. Lines show mean pHi of *n*_pH6.6_ = 35, *n*_pH6.6+CARI_ = 39, *n*_pH7.4_ = 45, *n*_pH7.4+CARI_ = 68, *n*_PDAC-CAF_ = 11, and *n*_PDAC-CAF+CARI_ = 22 cells from *N* = 3 mice each. (**F**) Quantification of resting pH_i_ of PSCs cultured at pH_e_6.6 (red) or pH_e_7.4 (blue) and CAFs (purple), respectively, derived from **E** (*n*_pH6.6_ = 74, *n*_pH7.4_ = 113, and *n*_PDAC-CAF+CARI_ = 41 cells from *N* = 3 mice each). (**G**) Scatter plot depicts the rate of Na^+^-independent pH_i_ recovery of PSCs cultured at pH_e_6.6 (red), pH_e_7.4 (blue), or CAFs (purple), derived from **E** (*n*/*N* see **F**). (**H**) Comparison of the rate of Na^+^-dependent pH_i_ recovery of WT PSCs cultured at pH_e_6.6 (red) or pH_e_7.4 (blue), or CAFs (purple) as explained in **E** (*n*/*N* see **E**). Statistical tests in **F**–**H** were performed with 1-way ANOVA with Tukey’s post hoc test.

Since the myofibroblastic phenotype switch occurs in PSCs at pH_e_7.4 but not at pH_e_6.6, we hypothesized that pH sensory and regulatory ion transporters are involved in the process. Thus, we evaluated our RNA-Seq data regarding the respective gene ontology term (GO:0015075; https://www.ebi.ac.uk/QuickGO/term/GO:0015075) (Figure 2A). We found 43 transporter genes differentially upregulated at pH_e_7.4 and 44 at pH_e_6.6 as well as a number of genes that were highly expressed in both groups (Supplemental Table 3). From these, we selected and further validated the expression of 5 transporter genes (Figure 2B) and 10 ion channel genes (Supplemental Figure 3) and compared them with the housekeeper genes *Ywhaz* and *18s* rRNA by means of quantitative PCR (qPCR). We used freshly isolated PSCs as well as PSCs cultivated at pH_e_7.4 or pH_e_6.6 for 5 days (Figure 2B; *n* = 6 from *N* = 3 mice). Notably, the Na^+^/H^+^ exchanger NHE1 (encoded by *SLC9A1*) has more mRNA expression in PSCs than most other pH-regulatory proteins under all investigated conditions. Because of its pathophysiological relevance in cancer (23) and good druggability with small molecules such as cariporide (24), we decided to further investigate this protein.

To explore the subcellular localization of NHE1 in vitro, we performed immunocytochemistry. Freshly isolated PSCs derived from healthy WT mice — i.e., quiescent PSCs — expressed NHE1 predominantly intracellularly (Figure 2C). However, NHE1 translocated to the plasma membrane of PSCs from healthy WT mice already after 24 hours of culturing in either pH_e_6.6 (iPSC) or pH_e_7.4 (myPSC). Similarly, CAFs freshly isolated from murine PDAC (KPfC) expressed NHE1 also mainly in their membrane.

Western blot analysis confirms our immunocytochemistry results. NHE1 was highly expressed in both freshly isolated and cultured PSCs as well as in PDAC-derived CAFs (Figure 2D). However, there were distinct differences: freshly isolated PSCs expressed the small (~80 kDa) intracellular, nonglycosylated NHE1 protein, whereas PSCs cultured for 120 hours and tumor-derived CAFs expressed the larger (~100 kDa) glycosylated, plasma membrane-residing NHE1.

We next focused on NHE1 functionality. We revealed its activity by measuring the pH_i_ recovery following an NH_4_^+^ prepulse (25) (Figure 2E). When PSCs from WT mice are cultured in vitro for 72 hours, the pH_i_ closely follows the pH_e_ (Figure 2F). PSCs kept at pH_e_6.6 have a pH_i_ of pH_i_ 6.60 ± 0.01 (*n* cells/*N* mice = 74/3). In contrast, the pH_i_ of PSCs cultured at pH_e_7.4 was pH_i_ 7.10 ± 0.02 (*n* cells/*N* mice = 113/3). Similarly, the pH_i_ of CAFs freshly isolated from KPfC-mice (cultured at pH_e_7.4) was pH_i_ 7.11 ± 0.01 (*n* cells/*N* mice = 41/3). The NH_4_^+^ prepulse protocol revealed almost no Na^+^-independent pH recovery in any of these cells (Figure 2G) (pH_e_6.6: 0.040 ± 0.004 pH unit/min, *n* cells/*N* mice = 156/5; pH_e_7.4: 0.060 ± 0.003 pH unit/min, *n* cells/*N* mice = 135/5; PDAC-CAF: 0.015 ± 0.005 pH unit/min, *n* cells/*N* mice = 41/3). In contrast, pH_i_ immediately recovered upon readdition of extracellular Na^+^. The rate of pH_i_ recovery was faster in PSCs cultured at pH_e_7.4 than in PSCs cultured at pH_e_6.6 (Figure 2H) (pH_e_7.4: 0.38 ± 0.05 pH unit/min, *n* cells/*N* mice = 45/3; pH_e_6.6: 0.11 ± 0.01 pH unit/min, *n* cells/*N* mice = 35/3; *P* < 0.0001). The pH recovery was primarily due to the action of NHE1, as inhibition of NHE1 with cariporide (10μM) almost completely abolished pH_i_ recovery (pH_e_6.6: 0.019 ± 0.003 pH unit/min, *n* cells/*N* mice = 39/3; pH_e_7.4: 0.061 ± 0.003 pH unit/min, *n* cells/*N* mice = 68/3). CAFs freshly isolated from tumor-bearing KPfC-mice also expressed a highly active NHE1, with the resting pH_i_ and the rate of NHE1-dependent recovery being similar to PSCs cultured at pH_e_7.4 (vehicle-treated KPfC-mice: pH recovery: 0.44 ± 0.09 pH unit/min, *n* cells/*N* mice = 11/3). This also applies to CAFs isolated from another PDAC mouse model that is driven by κB-Ras1 deficiency (26) (Supplemental Figure 4, A and B, *n*_pH6.6_ = 111, *n*_pH7.4_ = 94 cells from *n* = 5 mice).

RNA-Seq and qPCR analyses indicate that the Na^+^- HCO_3_^–^ cotransporter NBC1 (*Slc4a4*) and the monocarboxylate transporter 4 (*Slc16a3*) and numerous other transporters are also expressed in PSCs that could complement and/or compensate for blocked NHE1 activity. To rule out this possibility, we performed pH_i_ measurements in CAFs derived from vehicle-treated KPfC-mice in a CO_2_/HCO_3_^–^ buffered environment where HCO_3_^–^ transporters are active (Supplemental Figure 4C). We found that Na^+^-dependent pH recovery was primarily due to the action of NHE1 (Supplemental Figure 4, D and E; pH recovery: 0.68 ± 0.04 pH unit/min, *n* cells/*N* mice = 30/3). In summary, NHE1 acted as the major acid extruder in PSCs and CAFs, and its inhibition with cariporide led to intracellular acidification of in vitro cultured PSCs and ex vivo PDAC-derived CAFs.

### Lack of acidity facilitates YAP-1–mediated mechanotransduction and myofibroblastic PSC differentiation.

**Figure 3:**
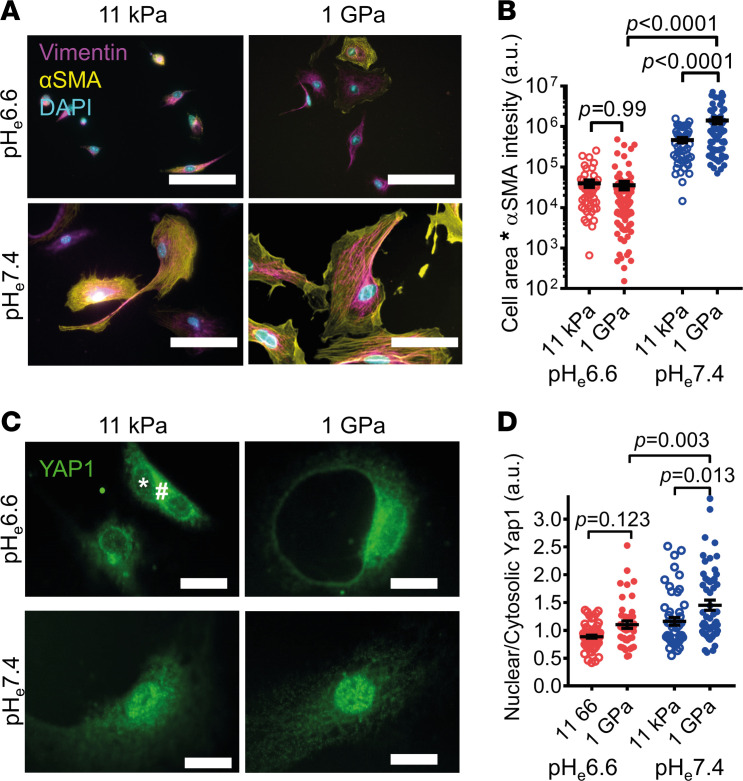
PSC mechanotransduction mediated by YAP1 is inhibited at acidic pH_e_. (**A**) Immunocytochemistry images of PSCs cultured on substrates of different stiffnesses at pH_e_6.6 (top) and pH_e_7.4 (bottom). The activation marker αSMA (yellow) and the general stellate cell marker vimentin (magenta), as well as nuclei (cyan), are labeled. Scale bar: 50 μm. (**B**) Cell area multiplied with mean αSMA fluorescence intensity was taken as a readout of myofibroblastic PSC phenotype on hydrogels with 11 kPa stiffness or on glass (1 GPa). *n*_11kPa_ = 65, *n*_1GPa_ = 170 cells from *N* = 3 mice. (**C**) Representative immunofluorescence images of YAP1 in PSCs (green) under the indicated cell culture conditions. YAP1, when translocated from the cytosol (#) into the nucleus (*), initiates transcription. Scale bar: 50 μm. (**D**) The ratio of nuclear/cytosolic YAP1 fluorescence intensity was determined as a readout of YAP1-mediated signal transduction. *n*_11kPa-pH6.6_ = 68, *n*_11kPa-pH7.4_ = 51, *n*_1GPa-pH6.6_ = 43, *n*_1GPa+pH7.4_ = 63 cells from *N* = 3 mice each. Statistical tests in **B** and **D** were performed with 1-way ANOVA with Tukey’s post hoc test.

Next, we aimed to get mechanistic insight into how cellular pH alkalinization results in the PSC phenotype switch. It is known that PSCs are quite susceptible to mechanical stimuli and express αSMA and, thus, exhibit a myofibroblastic phenotype predominantly on a rigid substrate (27, 28). We hypothesized that this major pathway of myPSC differentiation may be influenced by environmental pH. Therefore, we plated freshly isolated WT murine PSCs onto hydrogels with varying stiffness (Figure 3A) and cultured them at pH_e_7.4 or pH_e_6.6 for 72 hours. Cells were larger and had more αSMA (derived from cell area ***×*** αSMA intensity) when plated on the 1 GPa glass substrate than on the 11 kPa hydrogel surface (Figure 3B) (1 GPa: 335,493 ± 28,248 a.u., *n* cells/*N* mice = 170/3; 11 kPa: 88,304 ± 12,031 a.u., *n* cells/*N* mice = 65/3; *P* < 0.0001). This difference was not seen when culturing PSCs at pH_e_6.6 (1 GPa: 35,722 ± 7,685 a.u., *n* cells/*N* mice = 100/3; 11 kPa:39,577 ± 7,214 a.u., *n* cells/*N* mice = 51/3; *P* < 0.0001; Figure 3C). From this, we concluded that the myPSC but not the iPSC phenotype (PSCs cultured at pH_e_7.4 and pH_e_6.6, respectively) relied on substrate stiffness at least with respect to cell size and αSMA positivity.

We then investigated whether pH_e_ influenced mechanosignaling via YAP1, a well-characterized transcription factor in PSCs (29). Immunocytochemistry (Figure 3D) revealed that the nuclear/cytosolic ratio of YAP1 was higher in pH_e_7.4 than in pH_e_6.6, indicating increased YAP1-mediated transcription in myPSCs as compared with iPSCs (Figure 3D) (1 GPa, pH_e_7.4: 1.5 ± 0.1, *n* cells/*N* mice = 63/3; pH_e_6.6: 1.1 ± 0.1, *n* cells/*N* mice = 43/3; *P* = 0.018). Overall, these data point out that the YAP1-mediated mechanosignaling was pH dependent and not utilized in iPSCs kept in an acidic environment.

### Acidic pH_e_ partially alters myPSC phenotype only in the presence of cariporide.

**Figure 4:**
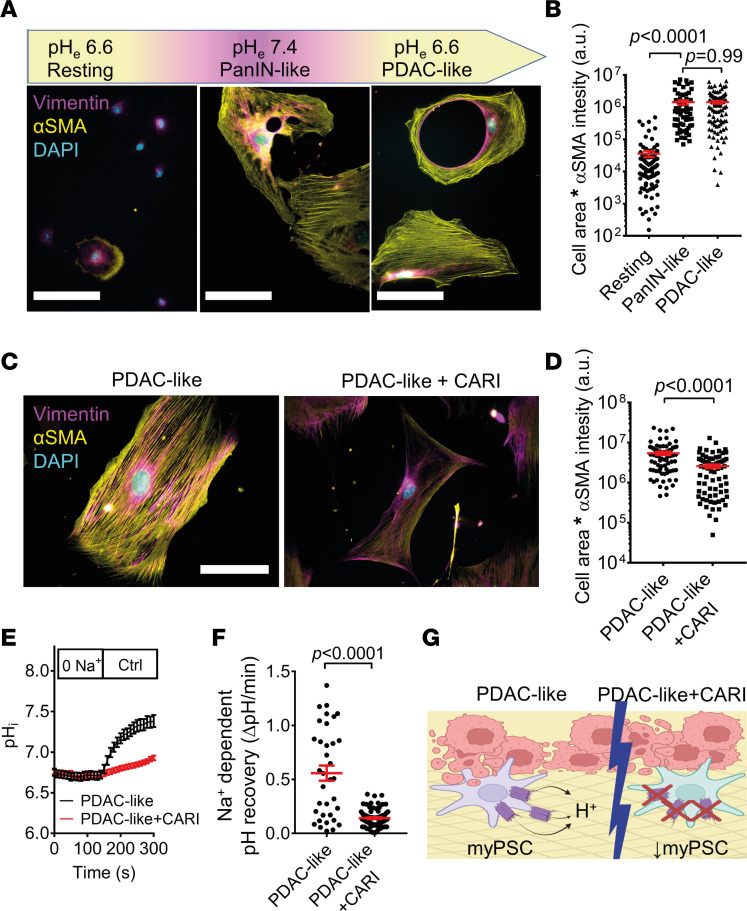
The myofibroblastic phenotype of activated PSCs is partially reversed by cariporide but not by acidic pH_e_ alone. (**A**) After culturing PSCs at pH_e_6.6 for 72 hours, medium was changed to pH_e_6.6 (Resting) or to pH_e_7.4 (PanIN-like) for another 72 hours. Lastly, the medium of pH_e_7.4-incubated cells was reacidified to pH_e_6.6 for another 72 hours (PDAC-like). Representative images of PSCs stained for αSMA (yellow), vimentin (magenta), and DAPI (cyan) are shown below each condition. Scale bar: 50 μm. (**B**) Scatter plot shows cell areas multiplied by αSMA fluorescence staining intensity (logarithmic scale) under conditions described in **A**. *n*_Resting_ = 100, *n*_PanIN-like_ = 64, and *n*_PDAC-like_ = 94; *N* = 3 mice. (**C**) Immunocytochemistry of PDAC-like PSCs in the absence (left) or presence (right) of cariporide (CARI). Scale bar: 50 μm. (**D**) Scatter plot shows cell areas multiplied by αSMA fluorescence intensity (logarithmic scale) under conditions described in **C**. *n*_PDAC-like_ = 74, *n*_PDAC-like+CARI_ = 74; *N* = 3 mice. (**E**) Intracellular pH measurements of PSCs where culture medium is reacidified after activation (pH_e_7.4 → pH_e_6.6, PDAC-like). Intracellular pH was acidified temporarily by applying the NH_4_^+^ prepulse technique, as shown in Figure 2. NHE1-dependent pH recovery can be observed when cells are superfused with Na^+^-containing solution (Ctrl) without (black) or with cariporide (red). Lines indicate the mean pH_i_ of *n*_PDAC-like_ = 35, *n*_PDAC-like+CARI_ = 79 cells from *N* = 3 mice. (**F**) Comparison of the rate of Na^+^-dependent recovery of PSCs in the absence or presence of cariporide derived from **E**. (**G**) Illustration of extended working hypothesis. In manifest PDAC, acidic pH_e_ fails to acidify pH_i_ because of NHE1-mediated H^+^ extrusion (left). Therefore, PSCs and CAFs remain myofibroblastic, ultimately promoting tumor desmoplasia. However, upon NHE1 inhibition with cariporide, PSCs and CAFs fail to counterbalance the acid stress, which disrupts the myofibroblastic phenotype (right). Statistical comparison in **B** was performed with 1-way ANOVA with Tukey’s post hoc test, whereas in **D** and **F**, statistical comparisons were performed with 2-tailed unpaired Student’s *t* tests. **G** was created with BioRender.com.

PSC phenotypes depend on the given stimulus (7, 30). Our results above indicate that environmental pH was crucial in PSC differentiation. However, in our initial experimental setting, we investigated a multitude of different pH-independent pathways by seeding cells from their native tissue environment onto plastic and into a cell culture medium. To test whether the sole removal of acidity was sufficient to induce differentiation of iPSC to myPSCs (e.g., through YAP-1–mediated mechanosignaling as shown above), we changed the medium pH of pH_e_6.6-cultured iPSCs, either to pH_e_6.6 (Resting) or to pH_e_7.4 (PanIN-like) after 72 hours (Figure 4A). Indeed, after just 3 days of culture at pH_e_7.4, PSCs differentiated from the iPSC to the myPSC phenotype as indicated by the drastic rise of αSMA expression (Figure 4B) (pH_e_6.6 → pH_e_6.6 [resting]: 35,722 ± 7685 a.u., *n* cells/*N* mice = 100/3; pH_e_6.6 → pH_e_7.4 [PanIN-like]: 1,414,669 ± 222,374 a.u., *n* cells/*N* mice = 64/3; *P* < 0.0001). Hence, we conclude that iPSCs cultured at pH_e_6.6 could further differentiate into myPSCs, with extracellular alkalinization (i.e., relief of extracellular acidity) being a sufficient stimulus.

We next tested whether PSCs (PanIN-like) can be reprogrammed from the myPSC to the iPSC phenotype by simply changing back the environmental pH to pH_e_6.6 (PDAC-like). This was not the case (Figure 4A). PSCs retained their myofibroblastic phenotype since αSMA positivity and cell size do not change (Figure 4B) (pH_e_7.4 → pH_e_6.6 [PDAC-like]: 1,421,502 ± 146,424 a.u., *n* cells/*N* mice = 94/3; *P* = 0.45). We reasoned that myofibroblastic PSCs retained their phenotype because of their pronounced NHE1 activity; reacidifying the environment alone would not alter pH_i_ anymore because cells could get rid of excess H^+^ rapidly through NHE1. To test this hypothesis, we applied cariporide with environmental reacidification (Figure 4C). In this “PDAC-like” pH shift, application of cariporide indeed affected the myPSC phenotype (Figure 4D) (PDAC-like+CARI: 2,575,631 ± 282,664 a.u., *n* cells/*N* mice = 74/3; PDAC-like: 5,421,677 ± 557,953 a.u., *n* cells/*N* mice = 74/3; *P* < 0.0001). This interpretation was supported by pH_i_ measurements (Figure 4E). NHE1-mediated pH_i_ recovery also occurred at a high rate in PSCs whose culture medium was reacidified after activation (pH_e_7.4 → pH_e_6.6) (Figure 4F) (Na^+^-dependent pH recovery: control: 0.56 ± 0.07 pH unit/min, *n* cells/*N* mice = 35/3; cariporide: 0.14 ± 0.01 pH unit/min, *n* cells/*N* mice = 79/3; *P* < 0.0001). These findings indicate that the NHE1 function maintained the myofibroblastic PSC phenotype in a harsh acidic tumor environment, while NHE1 inhibition partially disrupted it (Figure 4G).

### Adjuvant PDAC therapy with an NHE1 inhibitor reduces desmoplasia.

**Figure 5:**
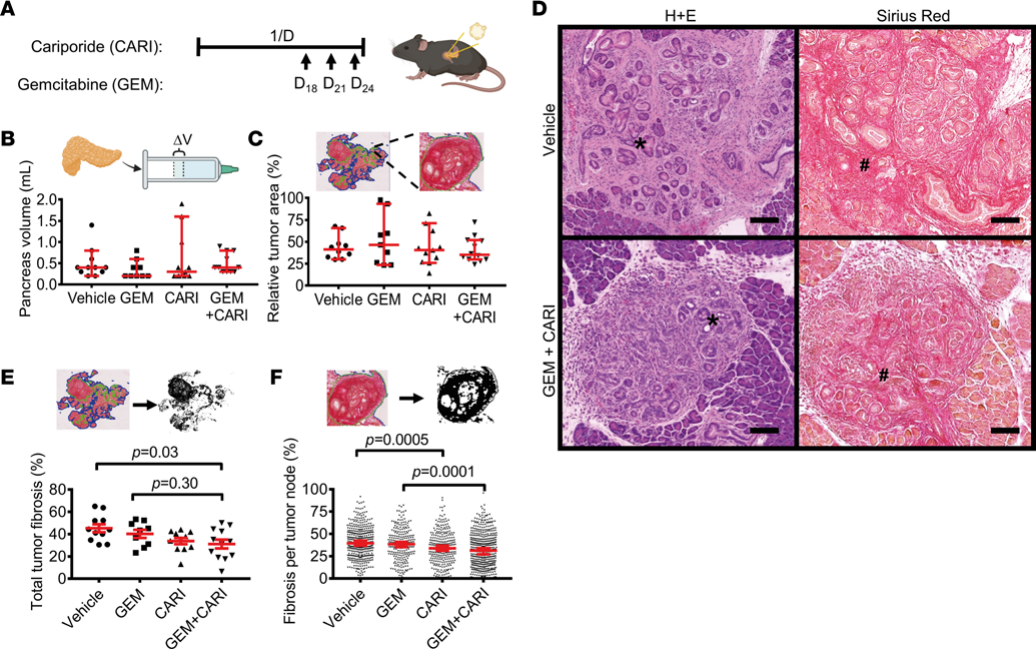
NHE1 inhibitor treatment leads to reduced desmoplastic reaction in murine PDAC. (**A**) Schematic representation of the 4-week–long treatment protocol of KPfC mice. Treatment started at the age of week 20. Cariporide was applied daily (1/D), and gemcitabine (100 mg/kg i.p.) was coinjected with cariporide (3 mg/kg i.p.) on the days indicated by the arrows. (**B**) Total pancreas volume of KPfC mice after gemcitabine (GEM) or cariporide (CARI) monotherapy or gemcitabine + cariporide (GEM+CARI) combined chemotherapy. Inlet demonstrates that pancreas volume was measured via volume displacement. Data points depict individual pancreata; *N*_Vehicle_ = 11, *N*_GEM_ = 9, *N*_CARI_ = 11, *N*_GEM+CARI_ = 11. (**C**) Relative tumor area in histological KPfC tissue sections was obtained by dividing total tumor area by total tissue area after H&E staining. Data points depict individual pancreata; *N*_Vehicle_ = 11, *N*_GEM_ = 9, *N*_CARI_ = 11, *N*_GEM+CARI_ = 11. (**D**) Representative images of PDAC nodes (marked with *) after H&E and Sirius red stainings. The degree of fibrosis correlates with the area of Sirius red^+^ (marked in red, #) tissue neighboring the cancerous tissue. Scale bar: 100 μm. (**E**) Relative tumor fibrosis of each Sirius red–stained KPfC tissue section was determined by dividing the summed area of fibrosis within every tumor node (sum of thresholded black areas in every node in the inlet) by the summed area of the tumor nodes. Data points depict individual pancreata; *N*_Vehicle_ = 11, *N*_GEM_ = 9, *N*_CARI_ = 11, *N*_GEM+CARI_ = 12. (**F**) To obtain the fibrosis per tumor node, the area of Sirius red^+^ fibrosis (black thresholded area in the inlet) was divided by the total area of the respective tumor node. Data points depict individual tumor nodes; *n*_Vehicle_ = 400, *n*_GEM_ = 239, *n*_CARI_ = 279, *n*_GEM+CARI_ = 476. Data and statistical comparison in **B**, **C**, and **F** are shown as median ± 95% CI with Kruskal-Wallis statistical test with Dunn’s post hoc test, and in **E** as mean ± SEM with 1-way ANOVA with Tukey’s post hoc test. Inlets for **A** and **B** were created with BioRender.com.

If the inhibition of NHE1 decreased the myofibroblastic nature of PSCs, this would become evident by a reduced fibrosis in pancreatic cancer. We tested this idea in pancreatic tumor–bearing KPfC mice that harbored heterozygous loss of p53 and conditionally expressed mutant K-Ras (genotype *Kras^wt/LSL–G12D^ Tp53^fl/+^ PDX1-Cre^+^*) (31, 32). Cariporide was given as an adjuvant drug complementing the standard therapy with gemcitabine (28, 29). To mimic the clinical situation of patients with PDAC who usually suffer from a late diagnosis and therapy initiation, we started to apply cariporide at week 20. At that time point, most KPfC mice have already developed manifest PDAC (33). We treated the mice with 3 mg/kg cariporide i.p. daily for 1 month and initiated chemotherapy with gemcitabine at the end of this period (Figure 5A).

The macroscopic total volume of the pancreata (Figure 5B) and microscopic relative area of tumor lesions normalized to total tissue area (Figure 5C) did not differ between the gemcitabine + cariporide double treatment group and the vehicle-treated group (volume: gemcitabine + cariporide: 0.4 mL, 95% CI, 0.3–0.7 mL; *P* = 0.84, vehicle: 0.4 mL, 95% CI, 0.3–0.7 mL; relative tumor area: gemcitabine + cariporide: 35%, 95% CI, 32–50%, *n* = 12 mice; vehicle: 41%, 95% CI, 34–54%, *n* = 10 mice; *P* = 0.78). However, the histochemical analysis of fibrosis by means of Sirius red staining revealed a clear difference (Figure 5D). The total area of fibrosis in tumors relative to total tumor area was reduced by ~ 30% in mice treated with gemcitabine + cariporide compared with vehicle treatment (Figure 5E; gemcitabine + cariporide: 31 ± 4%, *n* = 12 mice; vehicle: 45 ± 4%, *n* = 11 mice; *P* = 0.03). To ensure that statistics were not biased by a few individual tumor nodes that were very large in size, we investigated the extent of fibrosis in each individual tumor node in every tissue section (Figure 5F). We confirmed the antifibrotic effect of cariporide. Cariporide decreased fibrosis when compared with vehicle treatment (cariporide: 34%, 95% CI, 31%–37%, *n* individual tumor nodes/*N* mice = 279/11; vehicle: 40%, 95% CI, 37%–42%, *n* individual tumor nodes/*N* mice = 400/11, *P* = 0.0005). Moreover, gemcitabine + cariporide treatment decreased fibrosis more than gemcitabine alone (gemcitabine + cariporide: 31%, 95% CI, 27%–34%, *n* individual tumor nodes/*N* mice = 476/12; gemcitabine: 38%, 95% CI, 35%–41%, *n* individual tumor nodes/*N* mice = 239/9 *P* = 0.0001). These findings point out that NHE1 inhibition was an effective adjuvant therapeutic strategy to counter excess fibrosis in PDAC. The following sections delineate whether the decrease in fibrosis was due to a direct effect on PSCs or, rather, an indirect effect mediated by other cell types such as immune cells.

### NHE1 orchestrates CAF activation in PDAC.

**Figure 6:**
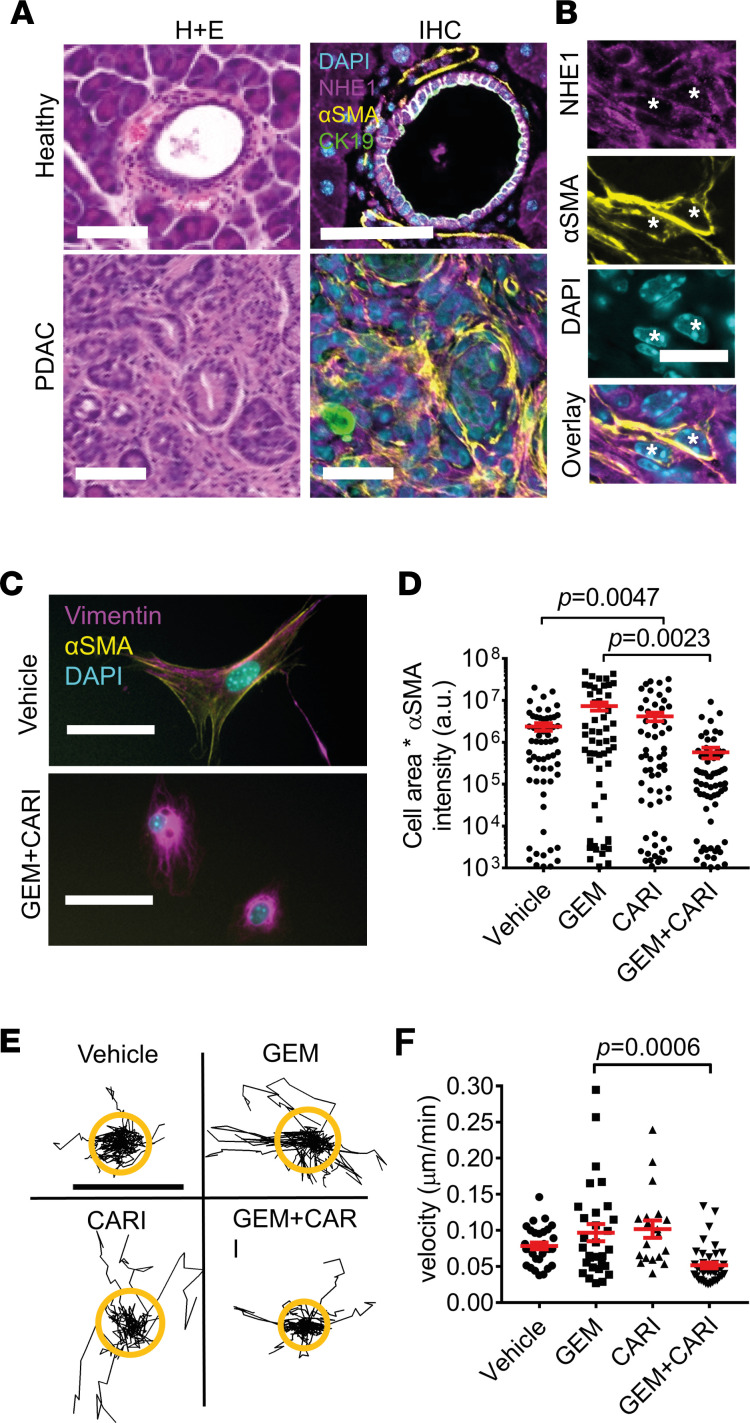
NHE1 orchestrates PDAC-derived CAF activation. (**A**) Representative H&E and IHC images of healthy and tumorous ducts. The colors of the IHC image indicate cell nuclei stained with NHE1 (magenta), αSMA^+^ PSCs and CAFs (yellow), DAPI (cyan), and CK19^+^ ductal or tumor cells (green). Scale bar: 40 μm. (**B**) αSMA^+^ cells (*) from **A** are depicted with higher magnification, which are also NHE1^+^. Scale bar: 20 μm (**C**) Immunocytochemistry of CAFs derived from KPfC mice after a 1-month treatment with vehicle (top) or gemcitabine + cariporide (bottom). Myofibroblast marker αSMA (yellow), the general mesenchymal marker vimentin (magenta), and nuclei (cyan) are labeled. Scale bar: 20 μm. (**D**) KPfC-derived CAF activation after therapy was assessed by multiplying cell area with the fluorescence intensity of αSMA. *n*_Vehicle_ = 61, *n*_GEM_ = 59, *n*_CARI_ = 63, *n*_GEM+CARI_ = 70 from *N* ≥ 3 mice. Note the logarithmic scale of the ordinate. (**E**) Trajectories of migrating KPfC-derived CAFs are shown by individual black lines. The treatment of the respective mice is indicated. Trajectories of the treatment groups are always normalized to common starting points. The radii of the orange circles highlight the mean translocation of cells in each population. Scale bar: 20 μm. (**F**) Mean cell migration velocities of individual CAFs were calculated from the trajectories in **C**. *n*_Vehicle_ = 30, *n*_GEM_ = 30, *n*_CARI_ = 19, *n*_GEM+CARI_ = 40 cells from *N* ≥ 3 mice. Statistical tests in **D** and **F** were performed with 1-way ANOVA with Tukey’s post hoc test.

We reasoned that the decreased fibrosis in the gemcitabine + cariporide treatment group was due to a decreased myCAF phenotype due to NHE1 inhibition. To ascertain whether PSCs express NHE1 in PDAC, we performed IHC (Figure 6A). We found that NHE1 was ubiquitously expressed in cell membranes in pancreatic cancer (Supplemental Figure 6). Indeed, it was expressed in αSMA^+^ CAFs (Figure 6B) but also CK19^+^ PDAC and ductal cells as well as in immune cells.

To investigate whether the myCAF phenotype was affected during gemcitabine + cariporide treatment, we isolated CAFs from KPfC mice after the treatments outlined in Figure 5A and immediately evaluated their phenotype from αSMA staining fluorescence intensity and cell size (Figure 6C). To verify the fibroblastic nature of the isolated cells, we showed by Western blot that only KPfC-derived tumor cells but not CAFs expressd a high level of mutated KRas G12D (Supplemental Figure 7). We found that CAFs derived from KPfC mice treated with gemcitabine + cariporide were markedly less myofibroblastic than those isolated from mice receiving a gemcitabine monotherapy (Figure 6D) (gemcitabine + cariporide; 592,059 ± 165,706 a.u., *n* cells/*N* mice = 70/4; gemcitabine: 7,339,572 ± 1,609,547 a.u., *n* cells/*N* mice = 59/3; *P* = 0.0023). When assessing cell area and αSMA fluorescence intensity separately (Supplemental Figure 5, A and B), we observed a decrease in cell area in gemcitabine + cariporide–treated CAFs compared with gemcitabine treatment (vehicle: 3,822 ± 412 μm^2^, *n* cells/*N* mice = 61/3; cariporide: 2,602 ± 255 μm^2^, *n* cells/*N* mice = 63/3; *P* = 0.038; gemcitabine: 3,308 ± 329 μm^2^, *n* cells/*N* mice = 59/3; gemcitabine + cariporide; 2,006 ± 245 μm^2^, *n* cells/*N* mice = 70/4; *P* < 0.0001). This decrease in cell area could be attributed to NHE1 inhibition and may be a reflection of a reduced cell volume (34).

Finally, as a global readout of CAF function, we assessed cell migration after the treatments (Figure 6E). CAFs derived from KPfC mice treated with gemcitabine + cariporide migrated more slowly than CAFs isolated from gemcitabine-treated mice (Figure 6F) (gemcitabine + cariporide: 0.05 ± 0.01 μm/min, *n* cells/*N* mice = 40/4; gemcitabine: 0.1 ± 0.01 μm/min, *n* cells/*N* mice = 30/3; *P* = 0.0006). We also recapitulated this finding by exposing untreated κB-Ras–deficient PDAC-derived CAFs to cariporide (Supplemental Figure 5C). In these cells, migration was also inhibited in the presence of 10 μM cariporide (Supplemental Figure 5D) (cariporide: 0.08 μm/min ± 0.01, *n* cells/*N* mice = 30/3; control: 0.11 ± 0.01 μm/min, *n* cells/*N* mice = 43/5; *P* = 0.025). To summarize, inhibition of the Na^+^/H^+^ exchanger NHE1 in WT and pancreatic tumor–bearing mice shifted CAF differentiation toward a less myofibroblastic phenotype.

### NHE1 inhibition enhances lymphocytic immune infiltration in KPfC mice.

**Figure 7:**
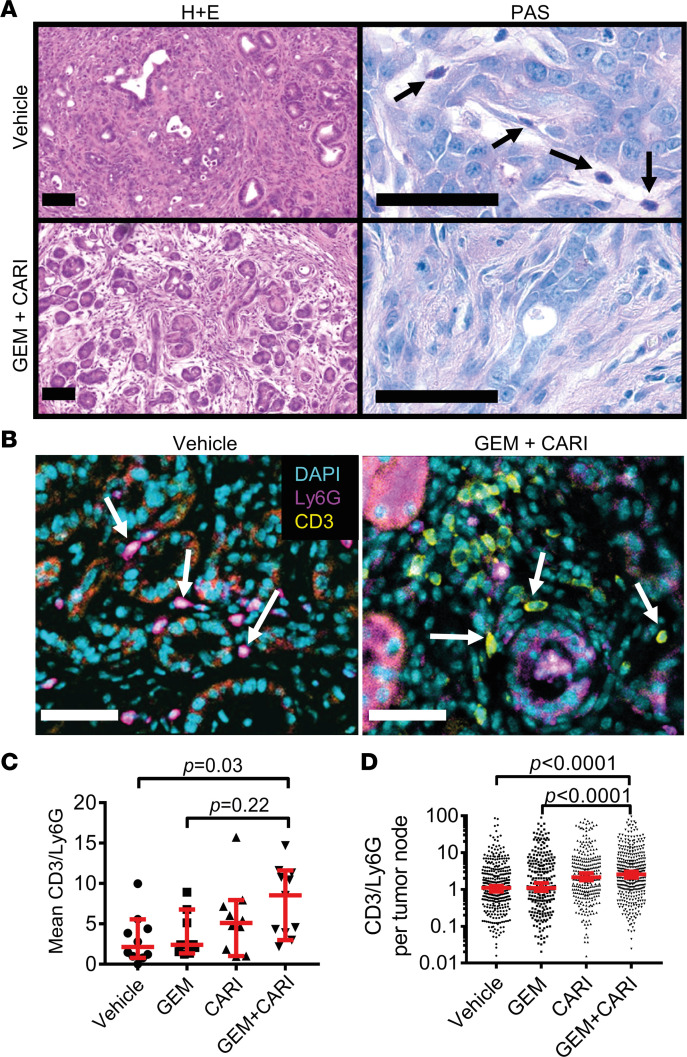
Lymphocyte/neutrophil ratio increases upon NHE1 inhibition in tumor sections of KPfC mice. (**A**) H&E (left) and PAS-stained KPfC mouse tissue sections after vehicle and gemcitabine + cariporide (GEM+CARI) therapy. Cells of innate immunity, such as neutrophils (arrows), utilize glycogen and are thus PAS^+^ (purple), in contrast to, for example, lymphocytes. Scale bar: 50 μm. (**B**) Representative IHC images stained for Ly6G^+^ neutrophils (magenta, arrows on left image), CD3^+^ lymphocytes (yellow, arrows on the right image), and nuclei with DAPI (cyan). Scale bar: 50 μm. (**C**) CD3/Ly6G ratio was assessed by dividing the number of CD3^+^ cells by the number of Ly6G^+^ cells in every tumor node. Data points depict the mean CD3/Ly6G ratio derived from each tumor node in individual mice; *N*_Vehicle_ = 10, *N*_GEM_ = 9, *N*_CARI_ = 10, *N*_GEM+CARI_ = 11 mice. (**D**) To obtain the CD3/Ly6G ratio per tumor node, the number of CD3^+^ cells was divided by the respective number of Ly6G^+^ cells in each tumor node. Data points depict individual tumor nodes; *n*_Vehicle_ = 386, *n*_GEM_ = 276, *n*_CARI_ = 301, *n*_GEM+CARI_ = 398. Data and statistical comparison in **D** and **E** are represented as median ± 95% CI using Kruskal-Wallis statistical test with Dunn’s post hoc test.

Above, we focused on NHE1 in PSCs and CAFs. However, it is known that a wide range of other cells, including PDAC cells, lymphocytes, and neutrophils, also express NHE1, as observable from Supplemental Figure 6 (18, 35–37). We, therefore, followed up the relevance of NHE1 in immune cell infiltration more closely after observing that the primarily periodic acid–Schiff^+^ (PAS^+^) infiltrates in vehicle- or gemcitabine-treated cohorts shifted to PAS^–^ ones in the cariporide or gemcitabine + cariporide treatment groups (Figure 7A and Supplemental Figure 8). Furthermore, the immune cell infiltration in cariporide-treated animals was often accompanied by a disruption of the architecture of tumor foci, which may indicate a more effective immune response. These observations suggest that NHE1 inhibition shifts the immune cell infiltrate from a largely innate immune cell–rich one to a more lymphocytic infiltration.

To confirm this idea, we further characterized each PDAC sample quantitatively with CD3 and Ly6G IHC staining, labeling T lymphocytes and neutrophils, respectively (Figure 7B). We focused our evaluation on the CD3/Ly6G ratio in PDAC lesions, specifying that a high lymphocyte/neutrophil ratio has a favorable prognostic value for patient survival (38). Indeed, when comparing the number of CD3^+^ and Ly6G^+^ cells in each tumor section, we found that the CD3/Ly6G ratio (Figure 7C) had increased by approximately 4 times in the gemcitabine + cariporide group as compared with the vehicle-treated group (gemcitabine + cariporide: 8.5, 95% CI, 4.9–10.6, *n* = 11 mice; vehicle: 2.2, 95% CI, 0.9–5.2, *n* = 9 mice; *P* = 0.03). Upon assessing the CD3/Ly6G ratio of each individual tumor node (Figure 7D), we found it to be 2.5-fold higher in the gemcitabine + cariporide–treated than in the gemcitabine-treated group (gemcitabine + cariporide: 2.6, 95% CI, 2.1–3.1, *n* individual tumor nodes/*N* mice = 398/11; gemcitabine: 1.1, 95% CI, 0.9–1.5, *n* individual tumor nodes/*N* mice = 276/9; *P* < 0.0001). We also noted that T cells not only accumulated in the periphery of individual tumor foci but also penetrated into the depth of the cancer tissue when mice were treated with gemcitabine + cariporide (Figure 7, A and B).

Taken together, our results indicate that the small-molecule inhibitor of NHE1, cariporide, targeted the PDAC stroma in vivo on at least 2 vital fronts; it disrupted the vicious cycle leading to marked fibrosis, and it shifted tumor immune cell infiltrate to a more lymphocytic one, consistent with a more potent antitumor response.

## Discussion

Our study was performed at the background of the unique pH landscape of the pancreas with the intermittent postprandial acidification of the interstitium that challenges all cells residing in the pancreatic stroma. Therefore, we studied how the inhibition of one of the major acid extruders, NHE1, affects the function and phenotype of PSCs, which are important cells in the pancreatic stroma. The ultimate question was whether targeting NHE1 affects PDAC progression. To this end, we showed that environmental acidosis is a crucial factor in keeping PSCs in the healthy organ in a nonmyofibroblastic state (Figure 1). This aligns well with our initial hypothesis that an intermittent acidosis after each meal would prevent the PSC phenotype change. The immunomodulatory-to-myofibroblastic phenotype switch was only induced after changing pH from pH_e_6.6 to pH_e_7.4 in vitro (Figures 1 and 4). These findings are consistent with the idea that the physiological intermittent acidity of the pancreas stroma acts like a “brake” to prevent premature PSC activation and excessive fibrosis. In pancreatic pathophysiology, removing this “acid brake” and the subsequent iPSC-to-myPSC phenotype switch favorably leads to ECM synthesis and wound healing in the short term. In the long term, however, reacidifying the environmental pH fails to revert the myPSC phenotype and terminate the fibrotic process, leading to excess fibrosis. Thus, depending on the context, interstitial/extracellular acidity acts like a double-edged sword. Thereby, the continuous supply of the stimulatory cancer cell secretome could make the difference in pancreatic cancer.

Based on our results, we argue that increased NHE1 activity, which is prominent in “PanIN-like” and “PDAC-like” myPSCs and tumor-derived CAFs (Figure 2), was a major determinant in maintaining the vicious cycle of the desmoplastic reaction. This assumption relies on the observation that PSCs only lost their myofibroblastic phenotype upon environmental reacidification when NHE1 was inhibited with cariporide (Figure 4). Further studies are needed to unravel whether this effect was due to decreased PSC activation or transdifferentiation to an immunomodulatory (or other) phenotype.

We could recapitulate this finding in vivo, where there was an apparent decrease in PDAC fibrosis (Figure 5) and myCAF phenotype (Figure 6) when applying the combined gemcitabine + cariporide treatment. These results imply that NHE1-inhibition may be a viable antifibrotic therapy in PDAC. However, antifibrotic drugs in cancer therapy are discussed controversially. On the one hand, complete elimination of fibrosis leads to enhanced invasiveness and more metastases (39). On the other hand, too much fibrosis creates a protective niche for tumor cells, allowing them to escape chemotherapy and immune response (40, 41). We propose that inhibiting NHE1 might be a compromise between not targeting the fibrotic PDAC stroma and its complete elimination, both of which are potentially harmful. Adjuvant therapy with cariporide reduced fibrosis and promoted lymphocyte infiltration (Figure 7) but did not lead to excess loss of ECM, which would promote tumor invasiveness (Figure 5). This complex response underpins that therapeutic NHE1 inhibition is not a purely antifibrotic therapy. Additional effects can, for example, be elicited by also targeting NHE1 in cancer cells (42).

In our view, we made 2 other important observations. We discovered that cariporide, in combination with gemcitabine, modulated tumor immune cell infiltration in favor of a CD3^+^ lymphocyte-rich immune response in KPfC mice (Figure 7). Based on our results, we propose a mechanism contributing to the neutrophil-to-lymphocyte switch in immune infiltration. Lymphocytes are unable to migrate through confined spaces when the mesh is too narrow for the nucleus to pass (43). Fewer myCAFs in the tumor reduce fibrosis, leading to an increased ECM mesh diameter, enabling lymphocytes to reach sites in the desmoplastic tumor that were too dense beforehand. On the other hand, neutrophil chemotaxis is impaired by cariporide (35). Taken together, it is plausible that both mechanical and biochemical cues lead to lymphocyte-rich immune response upon cariporide treatment. Other NHE1-regulated mechanisms, such as an altered metabolism, could also play a role. It has been recently shown that NHE1 inhibition promotes antitumor immune response in glioblastoma (36). Therapy of glioma-bearing mice with cariporide in combination with chemotherapy (temozolomide) induces metabolic rewiring and increases T-lymphocyte infiltration into the tumor through metabolic reprogramming.

Moreover, we found that changing environmental pH induced specific responses in PSCs. Acidification led to a dramatic upregulation of inflammation-related genes (Figure 1), whereas relative alkalization — i.e., relief of acidity — promoted cell proliferation and cell cycle (Figure 1, F–H). In addition, releasing the “acid brake” by alkalinization appears to be permissive for YAP1-mediated mechanotransduction (Figure 3) and subsequently for pathways involved in cell adhesiveness and proliferation (Figure 1 and Supplemental Figure 1). This result is reinforced by our earlier studies that a key mechanosensor in PSCs, Piezo1, is inhibited by acidic pH (44, 45) and that Piezo1 is coupled to the YAP/TAZ pathway (46). Also, altered cellular metabolism under differential mechanical stimuli in PDAC modulates energy production, which regulates cell adhesion, cytoskeletal dynamics, and ECM remodeling (47).

A key question regarding the therapeutic applicability of cariporide is whether inhibited NHE1 can be substituted by another acid-base regulating transporter in stellate cells or CAFs. This appears not to be the case; otherwise, cariporide therapy would not have shown the antifibrotic effects. Mechanistically, we found that the pH recovery after intracellular acidification happened in PSCs and CAFs primarily in a Na^+^-dependent manner in the absence and presence of HCO_3_^–^ (Figure 2). We did not find any functional evidence for Na^+^-independent pH regulators such as carbonic anhydrases or the vacuolar-type (V-type) ATPase in PSCs. After applying cariporide, the pH recovery became markedly slower in the presence of HCO_3_^–^ but was not completely abolished. This implies that, besides NHE1, the Na^+^/HCO_3_^–^ cotransporters such as NBC1 (*Slc4a4*) can further stabilize the pH_i_ in PSCs and CAFs. However, it has been recently shown that NBC1 in PDAC is rather expressed in epithelial tumor cells and regulates environmental HCO_3_^–^ accumulation, cellular glycolysis, and subsequent lactate release. Upon NBC1 inhibition, reduced lactate and increased HCO_3_^–^ in the TME improve CD8^+^ T cell–mediated immune response (37). This aligns with our results that revealed the increased lymphocyte/neutrophil ratio upon NHE1 inhibition (Figure 7). Whether there is an actual interplay between NHE1 and NBC1 that can be exploited therapeutically in PDAC is highly probable. NHE1 inhibition would primarily target CAFs, NBC1 inhibition would primarily target cancer cells, and both would favor lymphocytic immune response. Such a combined approach could target pancreatic cancer and stellate cells in a complementary way and result in an efficient modulation of the immune cell infiltrate. In summary, we argue that NHE1 function is not replaceable in PSCs but rather has a delicate interaction with other pH regulators culminating in an altered tumor immune response. These results support that cariporide should be explored as an adjuvant therapy in PDAC.

## Methods

### Animal experiments.

WT C57BL/6J mice were sacrificed for experiments involving in vitro–activated PSCs. Therapeutical studies with cariporide and gemcitabine were conducted in a blinded fashion with KPfC mice that harbored heterozygous loss of p53 and conditionally expressed mutant K-Ras (K-Ras^G12D^) from the endogenous locus in the pancreas (*Kras^wt/LSL–G12D^ Tp53^fl/+^ Pdx1-Cre^+^*) (31, 32). This is achieved by a Lox-SOP-Lox (LSL) cassette that prevents expression of the mutant K-Ras protein prior to Cre recombinase–mediated recombination and consequent excision of the STOP cassette that is controlled by the Pdx1 promotor (Pdx1-Cre) (48). Treatment with the NHE1 inhibitor cariporide or its vehicle (control group) started at the age of week 20 for 25 days (31, 49–51). Animals in the cariporide and gemcitabine + cariporide groups received daily i.p. injections with 3 mg/kg bodyweight cariporide (Medchemexpress) dissolved in 0.9% saline + 2% DMSO, with a total injection volume of 5.5 mL/kg bodyweight. Control animals were injected with 0.9% saline + 2% DMSO. Animals in the gemcitabine and caripoirde+gemcitabine group additionally received 3 dosages of 100 mg/kg bodyweight gemcitabine i.p. (Ely Lilly) at days 18, 21, and 24 (Summarized treatment protocol in Figure 5A). We used equal numbers of male and female animals in the 4 experimental groups.

As an additional validation of in vivo PSC activation, we isolated CAFs from κB-Ras–deficient mice, which were generated by crossing Pdx1-Cre and LSL-KRas^G12D^ with conventional κB-Ras and κB-Ras2–conditional KO mice. All mice were on a C57BL/6J background (48, 52). Mice were housed in individually vented cages (IVC) containing nesting material. Constant ambient temperature (22°C ± 2°C), constant humidity (55% ± 10%), and a 12-hour light/12-hour dark cycle were provided.

### Isolation of PSCs and CAFs.

PSCs and CAFs were isolated as described previously (44, 53). Briefly, murine pancreata were isolated from WT C57BL/6J mice and digested enzymatically with 0.1% collagenase P (Sigma-Aldrich, Merck KGaA) at 37°C for 25 min. We used a cube of 3 × 3 × 3 mm for isolating CAFs from KPfC or κB-Ras mouse-derived pancreata. After centrifugation with 200*g* at room temperature for 5 minutes, the homogenized tissue was resuspended in cell culture medium (DMEM/Ham F12 1:1, supplemented with 10% FCS and 1% penicillin/streptomycin; Sigma-Aldrich) and seeded onto FCS-coated tissue culture dishes, glass-bottom dishes, or hydrogel-coated glass-bottom dishes for 2 hours. CAFs derived from KPfC or κB-Ras–deficient mice were allowed to adhere for only 30 minutes, as we found that contamination with cancer cells became substantial after 2 hours. Afterward, nonadherent cells were vigorously washed off the tissue culture plate, resulting in a homogeneous PSC or CAF culture. Depending on the experiments, PSCs and CAFs were cultured for different time periods (2 hours, 24 hours, 3 days, 6 days, 9 days) with pH_e_6.6 or pH_e_7.4 media. To avoid trypsin-mediated PSC activation, PSCs and CAFs were used for experiments without passaging directly after isolation (54).

### RNA-Seq and qPCR.

For RNA-Seq, the Rneasy Mini Kit (Qiagen, 74104) was used to extract RNA from PSCs cultured for 120 hours at pH_e_6.6 or pH_e_7.4 according to the manufacturer’s instructions. The quality and quantity of isolated RNA were evaluated using the NanoDrop 2000. Libraries were prepared and sequenced (~20 M single reads per sample) using the Illumina Next-Seq 500 sequencing platform (High-Output Kit, 75 Cycles v2 Chemie) at the Genomics Core Facility (University Hospital Münster, Münster, Germany).

Subsequent bioinformatics processing and analysis were primarily carried out on the Galaxy platform (55). First, raw fastq files were aligned and mapped against the murine reference genome (mm10) with the HISAT2 v2.2.1 algorithm (RRID: SCR_015530) (56). Counts were subsequently extracted using featureCounts 2.0.1 (57), and differential expression of genes was analyzed using limma 3.50.1 (58), filtering out genes with CPM < 0.5 with a minimum of samples = 2. Principal component analysis shows low heterogeneity within biological replicates but a high degree of dissimilarity between different treatments (Supplemental Figure 1A). Features with FDR-adjusted *P* < 0.05 were declared significantly differentially expressed. The resulting gene lists were further processed with GSEA using fgsea 1.8.0 (59) and EGSEA 1.20.0 (60). RNA-Seq data are publicly available on the Gene Expression Omnibus (GEO; GSE223205).

For qPCR, RNA was prepared from freshly isolated WT-mouse derived PSCs and from PSCs cultured for 120 hours at pH_e_6.6 or pH_e_7.4, using TRIzol (Invitrogen) following the manufacturer’s instructions. cDNA was generated using SuperScript IV Reverse Transcriptase (Invitrogen) from 2 μg of RNA per reaction. qPCR was performed using TaqMan Gene Expression Master Mix (Thermo Fisher Scientific) with predesigned TaqMan probes on 96-well TaqMan Express Plates. qPCR was monitored using a QuantStudio 3 Real-Time PCR System (Thermo Fisher Scientific) using the cycling protocol of the master mix manufacturer. Data were analyzed using the QuantStudio Design and Analysis Software (Thermo Fisher Scientific). Since we found no published evaluation of housekeeping genes with respect to their pH_e_ sensitivity, we initially used 3 housekeeper genes for our assays. From *18s* rRNA, *Ywhaz*, and *Ppia*, *Ppia* was found to be expressed pH_e_ dependently and omitted from referencing by using the geNorm algorithm (RRID: SCR_006763) (61).

### Protein extraction and Western blot.

Total protein was extracted from freshly isolated WT mouse-derived PSCs, PSCs cultured for 120 hours at pH_e_6.6 or pH_e_7.4, and KPfC-derived CAFs using radioimmunoprecipitation assay (RIPA) buffer (Thermo Fisher Scientific) and 1% Complete Mini protease inhibitor (Roche). Protein concentration was assessed with Pierce BCA Protein Assay Kit (Thermo Fisher Scientific). Western blots of NHE1, vimentin, αSMA, KRas, and p53 were performed as described previously (62). In total, 15 μg of denatured total cellular protein was applied to each lane of 4%–15% Mini-PROTEAN TGX Stain-Free Protein Gels (Bio-Rad) for electrophoresis at 80 mV. After overnight transfer to PVDF membranes at 4°C, we detected the total amount of protein on the membrane following the manufacturer’s instructions. Next, we blocked the membrane with PBS containing 5% skim milk for 1 hour and then incubated the blots with primary antibodies against NHE1 (611775, RRID: AB_399261, 1:1,000, BD Bioscience), p53 (10442-1-AP, RRID: AB_2206609, 1:1,000, Proteintech), αSMA (A2547, RRID: AB_476701, 1:500, Sigma-Aldrich, Merck KGaA), vimentin (10366-1-AP, RRID: AB_2273020, 1:1,000, Proteintech), and G12D mutant–specific KRas antibody (14429, RRID: AB_2728748, 1:500, Cell Signaling Technology) overnight at 4°C. After washing 3 times with PBS, we applied HRP-conjugated goat anti-mouse secondary antibody (1:10,000, goat anti–mouse IgG H&L, ab6708, RRID: AB_956005, Abcam). Chemiluminescence was detected using a Chemidoc MP detection system (Bio-Rad), and band intensities were evaluated with the ImageLab software (Bio-Rad).

### Histology, IHC, and immunocytochemistry.

For histology and IHC, pancreata were fixed in 4% paraformaldehyde, embedded in paraffin, and then cut into 2 μm sections with a RM2125 microtome (Leica). Afterward, sections were deparaffinized with xylene, rehydrated in a stepwise manner, and stained with H&E or PAS (Biozol) and Sirius red (Roche) according to the manufacturer’s instructions. Representative images were taken with AxioImager (Zeiss) and subsequently processed with ImageJ (RRID: SCR_003070). For scanning whole tissue slices, the Leica SCN400 scanner was used with the Leica SCN400 Client software (Leica). Analysis of whole tissue scans was performed with QuPath software in a blinded fashion (RRID: SCR_018257) (63). First, a pixel classifier was trained to distinguish Sirius red^+^ fibrosis from nonfibrotic tissue using training images derived from multiple different tissue sections. The quality of the pixel classification algorithm was compared with the manual classification of fibrosis, and training was stopped when the automatic classification was at least as accurate as the manual classification. Then, individual tumor nodes were manually annotated on Sirius red–stained slices. Next, the pixel classification algorithm was applied to all Sirius red–labeled sections, resulting in the absolute and relative areas of Sirius red^+^ fibrosis in each tumor node as an output.

For IHC stainings, antigen retrieval was done using 10 mM sodium citrate buffer (pH 6.0), followed by blocking in 1% BSA-containing PBS for 1 hour (Sigma-Aldrich). Slides were stained with primary antibodies in a humidified chamber at 4°C overnight. Primary antibodies included the following: goat anti-CK19 (sc-33111, RRID: AB_2234419, 1:100, Santa Cruz Biotechnology), rabbit anti-NHE1 (HPA052891, RRID: AB_2681981, 1:200, Atlas Antibodies), and Alexa Fluor 488–conjugated mouse anti-αSMA (53-9760-82, RRID: AB_476701, 1:600, Thermo Fisher). In case of immune cell staining, we used: rabbit anti-CD3 (ab5690, RRID:AB_305055, 1:200, Abcam) and rat anti-Ly6G (MAB1037, 1:100, R&D Systems). After washing 3 times in PBS, the following secondary antibodies were used: donkey anti–rabbit Alexa Fluor 488 (711-545-152, RRID: AB_2313584, 1:1,000, Jackson ImmunoResearch) and donkey anti–goat-Cy3 (705-165-003, RRID: AB_2340411, 1:1,000, Jackson ImmunoResearch). Slides were mounted in DAKO mounting medium (Agilent) with 0.001% DAPI (Sigma-Aldrich, Merck KGaA) and covered with coverslips. For representative images, confocal microscopy was performed on a Leica DMI 6000 setup (Leica) in the Las-X software with 405 nm, 488 nm, 514 nm, and 633 nm lasers with a 630***×*** total magnification. Afterward, images were processed using ImageJ (NIH).

An inverted Nikon ECLIPSE Ti2 widefield microscope (Nikon) equipped with a triggered and calibrated Lumencor Spectra III LED light source, a motorized stage, 4***×*** (NA = 0.2) and 20***×*** (NA = 0.75) air objectives, and a Photometrics Prime 95B sCMOS camera was used in a semiautomated manner to acquire the Ly6G (Alexa Fluor 647), CD3 (Cy3), and the nuclear (DAPI) signals of the 5 μm–thick tissue sections in their entirety at a 550 nm per-pixel resolution. For greater efficiency, the microscope slides were positioned in a custom-built slide holder capable of carrying 4 slides simultaneously. The Nikon NIS Elements (ver. 5.21.03) JOBS acquisition automation was set up to first record a low-resolution (4***×*** objective) composite, DAPI overview image of all 4 slides. Next, outlines delineating the respective tissue section were generated, within which multiple points were distributed; at each point, the microscope automatically identified the respective *z* coordinate corresponding to the tissue in-focus plane. The resulting *z* coordinates were interpolated to generate a tissue section–specific focus map, along which the final, overlapping, multichannel images were acquired and ultimately stitched into a single image depicting the entire tissue section. The acquisition speed was enhanced by having the camera directly trigger the respective excitation wavelengths (635, 555, and 390 nm), utilizing a multibandpass filter cube and short (30 ms) exposure times. For subsequent image analysis, the resulting Nikon.nd2 files were batch converted to the .ims format (hierarchical data format 5 based) with the Imaris File Converter ver. 9.8.2 (Bitplane). All samples were acquired in a blinded fashion using the same acquisition settings.

IHC was evaluated in a blinded manner in QuPath (63). First, an object classification algorithm was trained from training images derived from multiple different tissue sections to separately detect CD3^+^ and Ly6G^+^ cells. The training was stopped when the manual classification was similar to automatic classification. Next, every tumor node in each tissue section was manually annotated, followed by segmentation of each individual cell in the tumor nodes using the DAPI channel. Finally, cells were classified as CD3^+^ and Ly6G^+^ using the trained automatic cell classification algorithm, giving the number of CD3^+^ and Ly6G^+^ cells in each tumor node as output.

Immunocytochemistry was performed on PSCs isolated directly onto glass-bottom dishes, where they were ultimately visualized. Following washing cells with PBS, fixation, and permeabilization were performed with ice-cold methanol at –20°C for 5 minutes. Afterward, dishes were carefully washed 3 times with PBS and blocked with PBS containing 10% FCS. Primary antibodies against αSMA (A2547, RRID: AB_476701, 1:200, Sigma-Aldrich, Merck KGaA), vimentin (10366-1-AP, RRID: AB_2273020, 1:500, Proteintech), YAP1 (sc-101199, RRID: AB_1131430, 1:250, Santa Cruz Biotechnology), and NHE1 (611775, RRID: AB_399261, 1:200, BD Bioscience) were applied overnight at 4°C. After washing 3 times with PBS, Alexa Fluor 488–conjugated secondary antibodies against mouse (Invitrogen, 1:500) and Cy3-conjugated antibodies against rabbit (Invitrogen, 1:500) were applied at room temperature for 1 hour. Lastly, after washing 3 times with PBS, 0.01% DAPI was applied in 1 mL PBS, and cellular fluorescence was acquired within 24 hours.

### pH_i_ measurements.

Assessment of pH_i_ was conducted ratiometrically using the fluorescence indicator BCECF-AM (Invitrogen) as previously described (64). PSCs and CAFs were either superfused with HEPES-buffered or CO_2_/HCO_3_^–^ buffered Ringer’s solution. Prior to conducting pH_i_ measurements with HEPES-buffered Ringer’s solution, HCO_3_^–^ buffered cell culture medium was exchanged to the HEPES-buffered Ringer’s solution, and cells were allowed to adapt for 1 hour to the environment. HEPES-buffered Ringer’s solution had the following composition (in mM): 122.5 NaCl, 5.4 KCl, 1.2 CaCl_2_, 0.8 MgCl_2_, 5.5 D-glucose, and 10.0 HEPES, titrated to pH 7.4 with NaOH. When performing pH_i_ measurements in the presence of CO_2_/HCO_3_^–^ buffered Ringer’s solution, all solutions contained 24 mM NaHCO_3_ instead of equimolar NaCl to maintain osmolarity, and solutions were continuously bubbled with 5% CO_2_ using the gas mixing system DIGAMIX SA27 (Wösthoff). The appropriate pH of solutions was regularly monitored by a Knick 766 electrode-based pH meter.

To study the NHE1-dependent pH recovery after acidification, NH_4_^+^ prepulse was performed (25). First, cells were perfused with 20 mM NH_4_^+^-containing Na^+^-free solution (in mM: 102.5 *N*-Methyl-D-glucamin hydrochloride, 20 NH_4_Cl, 5.4 KCl, 1.2 CaCl_2_, 0.8 MgCl_2_, 1 BaCl_2_, 5.5 D-glucose, and 10.0 HEPES, titrated to pH 7.4 with KOH) for 1 minute to alkalinize pH_i_. Afterward, PSCs were superfused with Na^+^-free solution (in mM: 122.5 *N*-Methyl-D-glucamin hydrochloride, 5.4 KCl, 1.2 CaCl_2_, 0.8 MgCl_2_, 5.5 D-glucose, and 10.0 HEPES, titrated to pH 7.4 with KOH) for 10 minutes to acidify pH_i_. Eventually, Na^+^ was added back with control Ringer’s solution for 5 minutes, and Na^+^-dependent recovery was observed in the presence of solvent (0.1% DMSO) or 10 μM cariporide. Lastly, for calibration purposes, cells superfused with a modified Ringer’s solution containing 1 μM nigericin (Sigma-Aldrich, Merck KGaA) (in mM: 125 KCl, 1 MgCl_2_, 1 CaCl_2_, and 20 HEPES, titrated to pH 7.5 and pH 6.5).

Data acquisition was performed with a setup consisting of a fluorescence microscope (Zeiss Axiovert 100), a high-speed shutter, and a polychromator (Visitron Systems), using dual excitation wavelengths of 440 nm and 490 nm and emission wavelength of 510 nm. Data analysis was performed using the Visiview 3.0 software (Visitron Systems). Fluorescence intensities were measured over the whole-cell area and corrected for background fluorescence. Subsequently, for each time point, the 440 nm_ex_/490 nm_ex_ fluorescence intensity ratios were calculated. Since the BCECF fluorescence ratio follows a linear trend between pH 6.5 and pH 7.5, 2-point calibration was performed using linear regression.

### Cell migration.

The migratory behavior of CAFs on a 2-dimensional substrate was assessed as previously described (44). Even though a 2-dimensional setting imposes nonphysiological constraints, it offers a reliable readout regarding the adhesive and migratory machinery of the cells (65). Briefly, CAFs originating from PDAC-bearing mice were seeded in 12.5 cm^2^ flasks that were precoated with ECM components resembling the desmoplastic stroma (66), containing the following components: 40 μg/mL laminin (Sigma-Aldrich, Merck KGaA), 40 μg/mL fibronectin (Sigma-Aldrich, Merck KGaAy), 800 μg/mL collagen I (Corning), 12 μg/mL collagen III (Corning), and 5.4 μg/mL collagen IV (BD Biosciences). Following cell adhesion overnight at 37°C containing 5% CO_2_, flasks were sealed air-tight and inserted into 37°C temperature-controlled chambers. In the chambers, cell motility was observed using an inverted microscope (Zeiss Axiovert 40, Zeiss) and recorded every other 15 minutes using the MicroCamLab 3.1 software (Bresser). From the recordings, cell contours were segmented using the Amira software (Thermo Fisher Scientific) and further processed to determine cell velocities (μm/min) from the displacement of the cell centroid over time (quantification detailed by Dieterich et al.; ref. 67). Cell translocation indicates the start-to-end net cellular displacement.

### Polyacrylamide hydrogel production and stiffness measurements.

Polyacrylamide gels were prepared using a chemical polymerization protocol (68). Glass-bottom dishes were defatted with EtOH and pretreated with 0.1M NaOH. Afterward, the glass was silanized with APTMS ([3-Aminoprpyl]trime-thoxysilane; Sigma-Aldrich) and subsequently treated with 0.5% glutaraldehyde at room temperature for 30 minutes. Different stiffness of polyacrylamide gels was achieved by applying different component concentrations, as detailed in Supplemental Table 1. After mixing the gel components, polymerization was initiated by 0.003% Tetramethylethylendiamine (TEMED) and 1% Ammoniumperoxodisulfate (APS). After mixing the solution, 8 μL solution was pipetted in the middle of the glass-bottom dish and immediately covered by a 15 mm diameter glass coverslip. Polymerization was allowed overnight at 4°C. Next, gels were treated with the photoactivated heterobifunctional crosslinker 0.05% Sulfo-SANPAH (Sigma-Aldrich, Merck KGaA) under 302 nm UV light for 15 minutes. After washing, gels were coated with the collagen-containing matrix used for cell migration studies.

Hydrogel stiffness was measured using a NanoWizard 3 NanoScience atomic force microscope (JPK Instruments AG). The cantilevers (Novascan Technologies), equipped with a spherical polystyrene bead of 10 μm diameter, were precalibrated, having spring constants of 0.03–0.04 N/m. The deflection sensitivity of the cantilever was calibrated before each measurement. Hydrogel stiffness measurements were conducted at 3 parts of the hydrogels, with 5 times probing the substrate at each point. All measured positions of the gel were at least 1 mm apart from each other to confirm gel homogeneity. The Young’s moduli were calculated from the measured force-distance curves using the JPK Data Processing software (Bruker Nano GmbH).

### Statistics.

Biological replicates (*N*) refer to the number of mice employed for each experimental series. *n* reflects the number of individual data points in any given experimental condition (e.g., number of cells, number of tumor foci). To calculate data distribution, the D’Agostino-Pearson test was applied. Normally distributed data are given as mean ± SEM; not normally distributed data are given as median ± 95% CI. For data following normal distribution, 2-tailed unpaired Student’s *t* tests or 1-way ANOVA with Tukey’s post hoc test were performed. When data did not follow a normal distribution, Mann-Whitney *U* test or nonparametric 1-way ANOVA-on-ranks (Kruskal-Wallis) statistical test with Dunn’s post hoc test was conducted. We used GraphPad Prism 7 (RRID: SCR_000306) for statistical analysis and data presentation. Statistical significance was assumed when *P* < 0.05.

### Study approval.

All animal experiments involving WT C57BL/6J mice, KPfC mice, and K-Ras^G12D^–expressing κB-Ras–deficient (*NKiras1^–/–^ NKiras2^fl/–^ Kras^wt/LSL–G12D^ Pdx1-Cre^+^*) mice were approved by the local authorities (LANUV).

### Data availability.

All raw data can be accessed in the Supporting Data Values file. Moreover, RNA-Seq data are publicly available on the GEO Omnibus (GSE223205). Further information and analytic methods that support the findings are available upon request from the corresponding author.

## Author contributions

Conceptualization was provided by ZP and AS. Methodology was provided by KN, SB, BF, SSC, SSA, IN, MW, KG, and MM. Investigation was provided by ZP, KN, SB, and MM. Visualization was provided by ZP, KN, and AS. Funding acquisition was provided by AO and AS. Project administration was provided by EW, AO, and AS. Supervision was provided by EW, AO, and AS. Writing of the original draft was provided by ZP, KN, MW, KG, and MM. Review and editing of the manuscript were provided by ZP, EW, AO, and AS.

## Supplementary Material

Supplemental data

Supplemental table 1

Supplemental table 2

Supplemental table 3

Supplemental table 4

Supporting data values

## Version 1 08/29/2023

In-Press Preview

## Version 2 10/09/2023

Electronic publication

## References

[1] https://www.cancer.org/content/dam/cancer-org/research/cancer-facts-and-statistics/annual-cancer-facts-and-figures/2023/2023-cancer-facts-and-figures.pdf.

[2] Amrutkar M, Gladhaug IP (2021). Stellate cells aid growth-permissive metabolic reprogramming and promote gemcitabine chemoresistance in pancreatic cancer. Cancers (Basel).

[3] Cruz-Monserrate Z (2014). Targeting pancreatic ductal adenocarcinoma acidic microenvironment. Sci Rep.

[4] Hofschröer V (2021). Ion channels orchestrate pancreatic ductal adenocarcinoma progression and therapy. Front Immunol.

[5] Wörmann SM (2014). The immune network in pancreatic cancer development and progression. Oncogene.

[6] Zhou Y (2019). Pancreatic stellate cells: a rising translational physiology star as a potential stem cell type for beta cell neogenesis. Front Physiol.

[7] Sunami Y (2020). Cellular heterogeneity of pancreatic stellate cells, mesenchymal stem cells, and cancer-associated fibroblasts in pancreatic cancer. Cancers (Basel).

[8] Sherman MH (2014). Vitamin D receptor-mediated stromal reprogramming suppresses pancreatitis and enhances pancreatic cancer therapy. Cell.

[9] Sunami Y (2021). Targeting and reprograming cancer-associated fibroblasts and the tumor microenvironment in pancreatic cancer. Cancers (Basel).

[10] Boron WF (2004). Regulation of intracellular pH. Adv Physiol Educ.

[11] Pethő Z (2020). pH-Channeling in cancer: how pH-dependence of cation channels shapes cancer pathophysiology. Cancers (Basel).

[12] Chaitman BR (2003). A review of the GUARDIAN trial results: clinical implications and the significance of elevated perioperative CK-MB on 6-month survival. J Card Surg.

[13] Mentzer RM (2008). Sodium-hydrogen exchange inhibition by cariporide to reduce the risk of ischemic cardiac events in patients undergoing coronary artery bypass grafting: results of the EXPEDITION study. Ann Thorac Surg.

[14] White KA (2017). Cancer cell behaviors mediated by dysregulated pH dynamics at a glance. J Cell Sci.

[15] Frontzek F (2014). Functional interdependence of NHE1 and merlin in human melanoma cells. Biochem Cell Biol.

[16] Stock C (2012). Is the multifunctional Na(+)/H(+) exchanger isoform 1 a potential therapeutic target in cancer?. Curr Med Chem.

[17] Stock C, Pedersen SF (2017). Roles of pH and the Na^+^/H^+^ exchanger NHE1 in cancer: from cell biology and animal models to an emerging translational perspective?. Semin Cancer Biol.

[18] Cardone RA (2015). A novel NHE1-centered signaling cassette drives epidermal growth factor receptor-dependent pancreatic tumor metastasis and is a target for combination therapy. Neoplasia.

[19] Pedersen SF (2017). Alternating pH landscapes shape epithelial cancer initiation and progression: focus on pancreatic cancer. BioEssays.

[20] Novak I (2013). Acid-base transport in pancreas—new challenges. Front Physiol.

[21] Ashley SW (1994). Pancreatic interstitial pH regulation: effects of secretory stimulation. Surgery.

[22] Öhlund D (2017). Distinct populations of inflammatory fibroblasts and myofibroblasts in pancreatic cancer. J Exp Med.

[23] Czaplinska D (2023). Crosstalk between tumor acidosis, p53 and extracellular matrix regulates pancreatic cancer aggressiveness. Int J Cancer.

[24] Harguindey S (2013). Cariporide and other new and powerful NHE1 inhibitors as potentially selective anticancer drugs - an integral molecular/biochemical/metabolic/clinical approach after one hundred years of cancer research. J Transl Med.

[25] Schwab A (2005). Functional role of Na+-HCO3- cotransport in migration of transformed renal epithelial cells. J Physiol.

[26] Beel S (2020). κB-Ras and Ral GTPases regulate acinar to ductal metaplasia during pancreatic adenocarcinoma development and pancreatitis. Nat Commun.

[27] Apte MV (1998). Periacinar stellate shaped cells in rat pancreas: identification, isolation, and culture. Gut.

[28] Bachem MG (1998). Identification, culture, and characterization of pancreatic stellate cells in rats and humans. Gastroenterology.

[29] Lachowski D (2018). FAK controls the mechanical activation of YAP, a transcriptional regulator required for durotaxis. FASEB J.

[30] Helms EJ (2022). Mesenchymal lineage heterogeneity underlies nonredundant functions of pancreatic cancer-associated fibroblasts. Cancer Discov.

[31] Hingorani SR (2003). Preinvasive and invasive ductal pancreatic cancer and its early detection in the mouse. Cancer Cell.

[32] Olive KP (2004). Mutant p53 gain of function in two mouse models of Li-Fraumeni syndrome. Cell.

[33] Veite-Schmahl MJ (2017). The mouse model of pancreatic cancer atlas (MMPCA) for classification of pancreatic cancer lesions: a large histological investigation of the Ptf1aCre/+;LSL-KrasG12D/+ transgenic mouse model of pancreatic cancer. PLoS One.

[34] Bera K (2022). Extracellular fluid viscosity enhances cell migration and cancer dissemination. Nature.

[35] Oster L (2022). Extracellular pH controls chemotaxis of neutrophil granulocytes by regulating LTB4 production and Cdc42 signaling. J Immunol.

[36] Hasan MN (2021). Blocking NHE1 stimulates glioma tumor immunity by restoring OXPHOS function of myeloid cells. Theranostics.

[37] Cappellesso F (2022). Targeting the bicarbonate transporter SLC4A4 overcomes immunosuppression and immunotherapy resistance in pancreatic cancer. Nat Cancer.

[38] Yang JJ (2015). Prognostic significance of neutrophil to lymphocyte ratio in pancreatic cancer: a meta-analysis. World J Gastroenterol.

[39] Rhim AD (2014). Stromal elements act to restrain, rather than support, pancreatic ductal adenocarcinoma. Cancer Cell.

[40] Mercanti L (2023). PDAC, the influencer cancer: cross-talk with tumor microenvironment and connected potential therapy strategies. Cancers (Basel).

[41] Hosein AN (2020). Pancreatic cancer stroma: an update on therapeutic targeting strategies. Nat Rev Gastroenterol Hepatol.

[42] Audero MM (2023). Acidic growth conditions promote epithelial-to-mesenchymal transition to select more aggressive pdac cell phenotypes in vitro. Cancers (Basel).

[43] Sadjadi Z (2020). Migration of cytotoxic T lymphocytes in 3D collagen matrices. Biophys J.

[44] Kuntze A (2020). Protonation of Piezo1 impairs cell-matrix interactions of pancreatic stellate cells. Front Physiol.

[45] Bae C (2015). Protonation of the human PIEZO1 ion channel stabilizes inactivation. J Biol Chem.

[46] Hasegawa K (2021). YAP signaling induces PIEZO1 to promote oral squamous cell carcinoma cell proliferation. J Pathol.

[47] Papalazarou V (2020). The creatine-phosphagen system is mechanoresponsive in pancreatic adenocarcinoma and fuels invasion and metastasis. Nat Metab.

[48] Gannon M (2000). Mosaic Cre-mediated recombination in pancreas using the Pdx-1 enhancer/promoter. Genesis.

[49] Johnson L (2001). Somatic activation of the K-ras oncogene causes early onset lung cancer in mice. Nature.

[50] Hingorani SR (2005). Trp53R172H and KrasG12D cooperate to promote chromosomal instability and widely metastatic pancreatic ductal adenocarcinoma in mice. Cancer Cell.

[51] Jackson EL (2001). Analysis of lung tumor initiation and progression using conditional expression of oncogenic K-ras. Genes Dev.

[52] Oeckinghaus A (2014). κB-Ras proteins regulate both NF-κB-dependent inflammation and Ral-dependent proliferation. Cell Rep.

[53] Fels B (2016). Role of TRPC1 channels in pressure-mediated activation of murine pancreatic stellate cells. Eur Biophys J.

[54] Won JH (2011). Phenotypic changes in mouse pancreatic stellate cell Ca2+ signaling events following activation in culture and in a disease model of pancreatitis. Mol Biol Cell.

[55] Afgan E (2018). The Galaxy platform for accessible, reproducible and collaborative biomedical analyses: 2018 update. Nucleic Acids Res.

[56] Kim D (2015). HISAT: a fast spliced aligner with low memory requirements. Nat Methods.

[57] Liao Y (2014). featureCounts: an efficient general purpose program for assigning sequence reads to genomic features. Bioinformatics.

[58] Liu R (2015). Why weight? Modelling sample and observational level variability improves power in RNA-Seq analyses. Nucleic Acids Res.

[60] Alhamdoosh M (2017). Combining multiple tools outperforms individual methods in gene set enrichment analyses. Bioinformatics.

[61] Vandesompele J (2002). Accurate normalization of real-time quantitative RT-PCR data by geometric averaging of multiple internal control genes. Genome Biol.

[62] Bulk E (2017). K_Ca_3.1 channel inhibition leads to an ICAM-1 dependent increase of cell-cell adhesion between A549 lung cancer and HMEC-1 endothelial cells. Oncotarget.

[63] Bankhead P (2017). QuPath: open source software for digital pathology image analysis. Sci Rep.

[64] Stock C (2005). Migration of human melanoma cells depends on extracellular pH and Na+/H+ exchange. J Physiol.

[65] Tschumperlin DJ (2013). Fibroblasts and the ground they walk on. Physiology (Bethesda).

[66] Tian C (2019). Proteomic analyses of ECM during pancreatic ductal adenocarcinoma progression reveal different contributions by tumor and stromal cells. Proc Natl Acad Sci U S A.

[67] Dieterich P (2008). Anomalous dynamics of cell migration. Proc Natl Acad Sci U S A.

[68] Rheinlaender J (2020). Cortical cell stiffness is independent of substrate mechanics. Nat Mater.

