# Supplementary material for: Acid-base homeostasis orchestrated by NHE1 defines the pancreatic stellate cell phenotype in pancreatic cancer
Source: JCI Insight. 2023 Oct 9;8(19):e170928. doi: 10.1172/jci.insight.170928 (PMC10619433; doi:10.1172/jci.insight.170928)
Supplement: Supplemental data [file jciinsight-8-170928-s103.pdf]

Supplementary information

Acid-base homeostasis orchestrated by NHE1 defines pancreatic stellate cell phenotype in pancreatic cancer

Zoltán Pethő[1], Karolina Najder[1], Stephanie Beel[2], Benedikt Fels[1,3], Sandra Schimmelpfennig[1], Ilka Neumann[1], Sarah Sargin[1], Maria Wolters[4], Klavs Grantins[4], Eva Wardelmann[4], Miso Mitkovski[5], Andrea Oeckinghaus[2], Albrecht Schwab[1]

Affiliations

[1]Institute of Physiology II, University of Münster; Münster, Germany

[2]Institute of Tumor Biology, University of Münster; Münster, Germany

[3]Institute of Physiology, University of Lübeck; Lübeck, Germany

[4]Gerhard-Domagk-Institute of Pathology, University of Münster; Münster, Germany

[5]City Campus Light Microscopy Facility, Max Planck Institute for Multidisciplinary Sciences; Goettingen, Germany

[*]Corresponding author, pethoe@uni-muenster.de; Institute of Physiology II, University of Münster, Robert-Koch-Straße 27 B, 48149 Münster, Germany, Tel.: +492518355336, ORCID ID: 0000-0001-7057-4761

Supplementary Figures

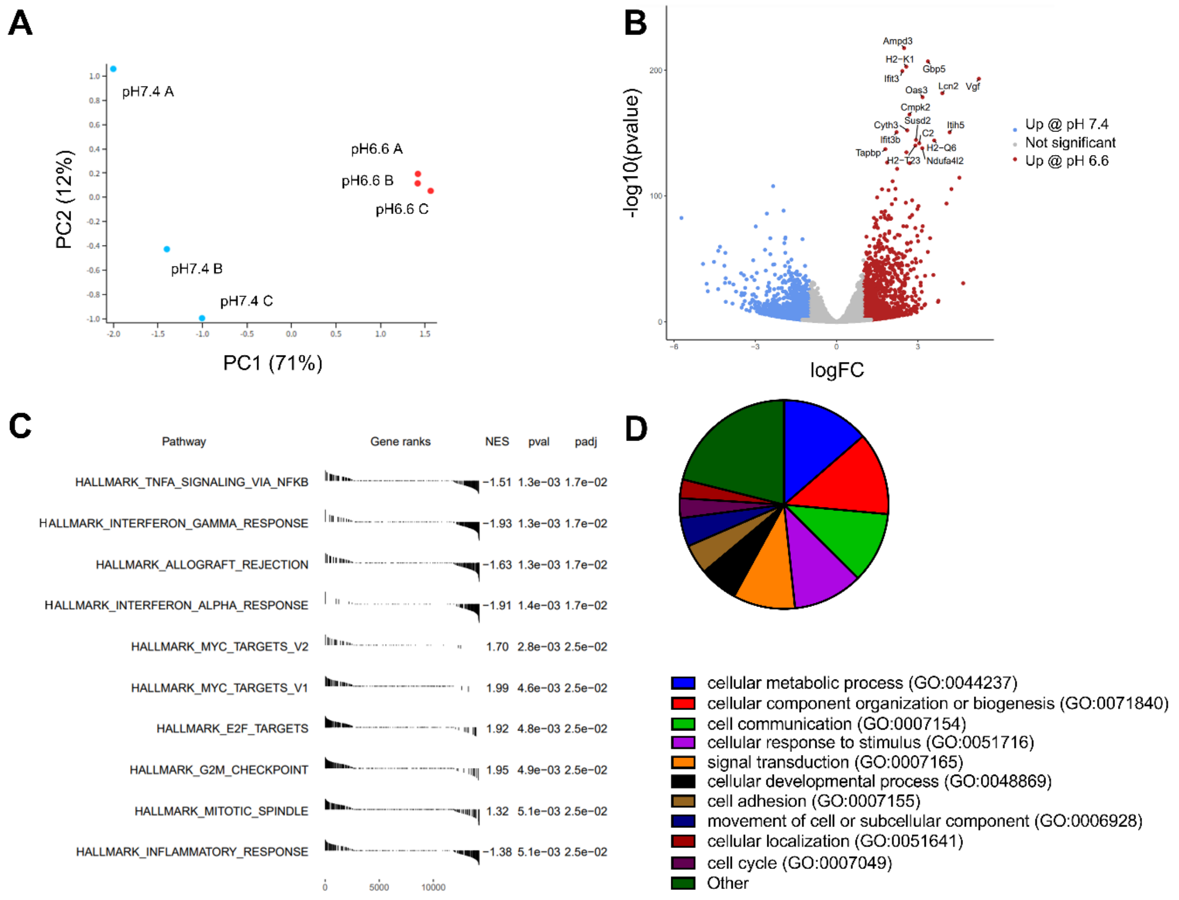

Supplementary Figure 1. Differentially expressed gene sets in PSCs at different pH_e

A Principal component analysis comparing PC1 and PC2 dimensions, with variance in brackets, in biological replicates (A-C) of PSCs cultured at pH_e 7.4 and 6.6, respectively. Briefly, PC1 largely describes differences between the two groups, whereas PC2 shows heterogeneity in the respective groups. **B** Volcano plot analysis of differentially expressed genes upregulated in PSCs cultured at pH_e7.4 (blue, n=2320) versus pH_e6.6 (red, n=2267). The top 20 differentially expressed genes are labeled. **C** FGSEA output shows hallmark gene sets of differentially expressed genes with respective gene ranks, normalized enrichment score (NES), raw and adjusted p values (pval and padj, respectively). **D** GSEA output of the top 10 biological processes coordinated by the differentially expressed genes.

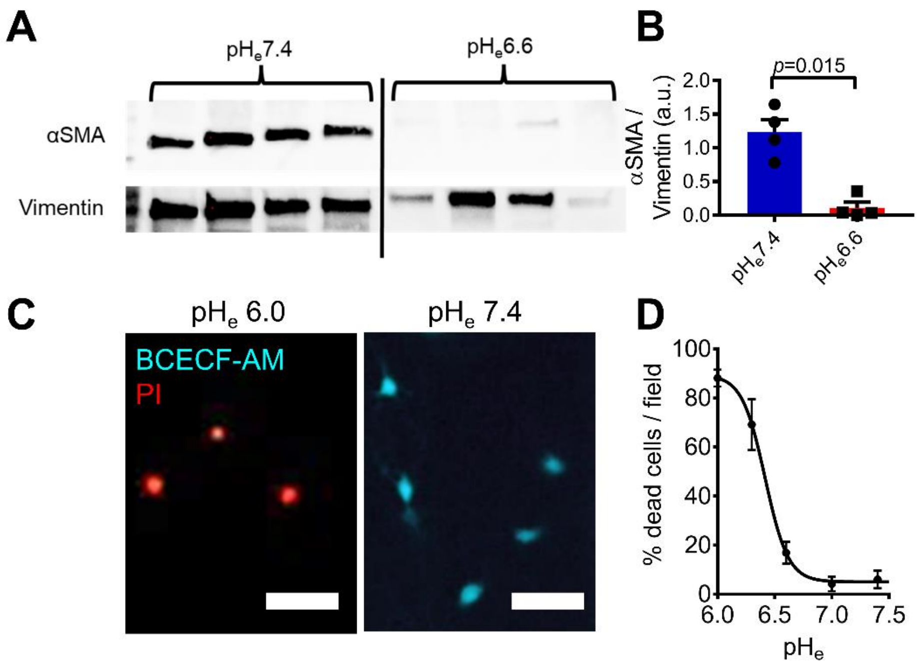

Supplementary Figure 2. Pancreatic stellate cells have reduced αSMA content and viability at low pH_e

A Western blot of αSMA (top lane) compared to vimentin (bottom lane) at pH_e 6.6 (left) and pH_e7.4 (right) from N=4 mice. **B** Quantification of Western blot depicted in A). **C** Representative immunofluorescence images and quantification for cell viability assessment by staining with the live cell marker BCECF-AM (cyan) and the dead cell marker propidium iodide (PI, red). Demonstrated are PSCs cultured at pH_e 6.0 (left) and pH_e7.4 (middle) Scale bar = 200 µm. **D** Percentage of dead cells in a field of view, measured at pH_e 6.0, pH_e 6.3, pH_e 6.6, pH_e 7.0 and pH_e 7.4. Data point indicates mean value from n=5 fields of view of N=5 mice. Note that cells are largely viable when $pH_e \geq 6.6$. Statistical test in (B) was performed with two-tailed unpaired Student's t-tests.

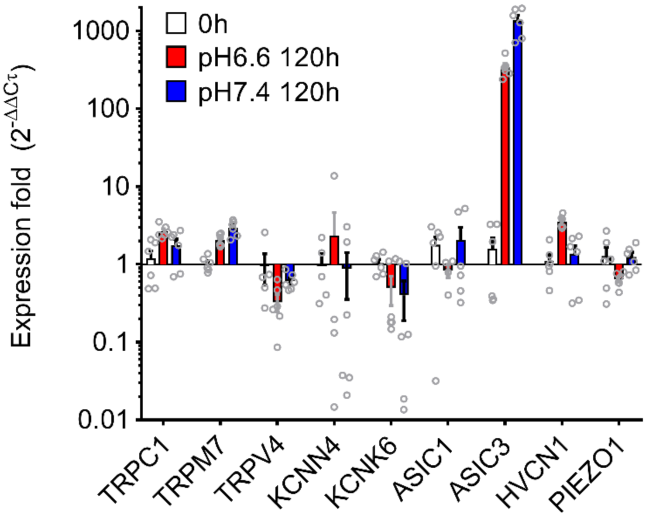

Supplementary Figure 3. Pancreatic stellate cells express multiple pH sensory ion channels

Bar chart shows RT-qPCR analysis gene expression of pH sensors and regulators compared to housekeeper genes YWHAZ and 18S rRNA in freshly isolated (0h, black) and cultured wild-type mouse-derived PSCs at pH_e7.4 (blue) or pH_e6.6 (red). Genes expression fold is compared to freshly isolated PSCs Data in are n=6 technical replicates from N=3 mice.

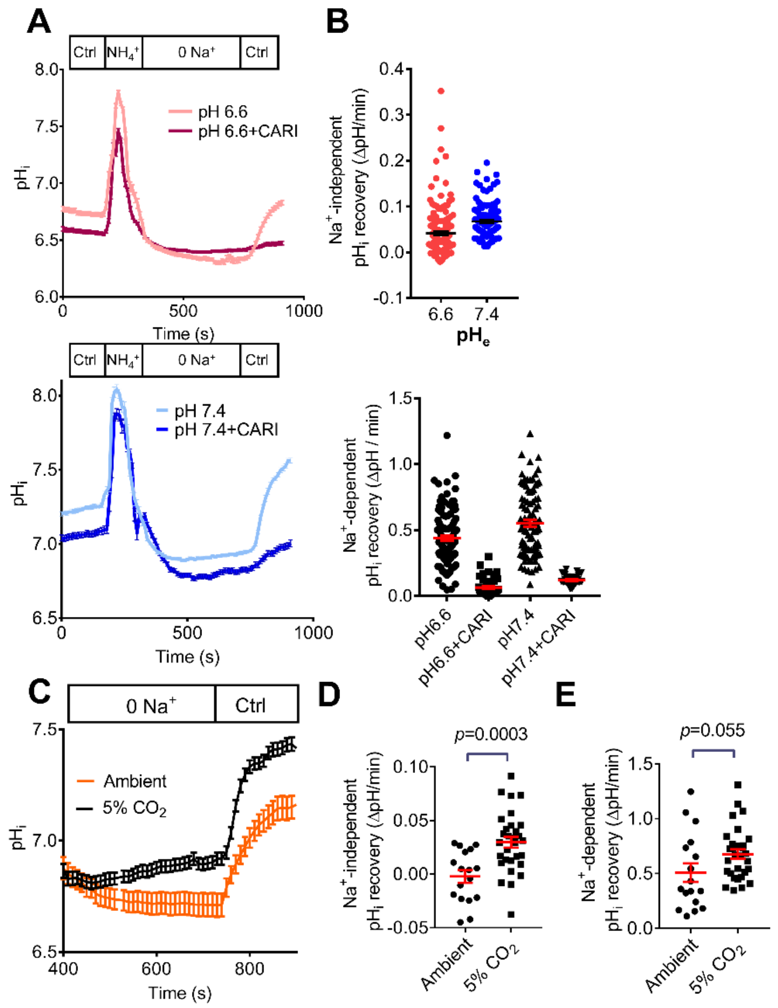

Supplementary Figure 4. NHE1 is functional in multiple PDAC-bearing mouse models under ambient conditions and 5% CO_2

A Intracellular pH recordings of κB-Ras1 deficient mouse-derived CAFs cultured at pH_e6.6 (top) and pH_e7.4 (bottom). Intracellular pH was acidified temporarily by applying the NH_4^+ prepulse technique. NHE1-independent pH_i recovery can be observed during the superfusion of Na^+-free “0 Na^+” solution. NHE1-dependent pH_i recovery can be observed in the last step (Ctrl) of the experiment, when cells were superfused with Na^+-containing solution. Cariporide was added to the superfusion at this step as indicated. Lines indicate mean pH_i of $n_{pH6.6}$=111, $n_{pH6.6+CARI}$=38, $n_{pH7.4}$=103, $n_{pH6.6+CARI}$=45 cells from N=3 mice. **B** Quantification of the rate of Na^+-independent (top) and Na^+-dependent (bottom) pH_i recovery of CAFs cultured at pH_e6.6 (red) or pH_e7.4 (blue), respectively, derived from panel (A). **C** Intracellular pH recordings of KPfC mouse-derived CAFs after vehicle, therapy under ambient environment (orange) compared to 5% CO_2 environment. Lines indicate mean pH_i of $n_{Ambient}$=19, $n_{5\%CO2}$=32 cells from N=3 mice. **D** Quantification of the rate of the Na^+-independent (bottom) pH_i recovery of CAFs cultured at conditions detailed in panel (C). **E** Quantification of the rate of the Na^+-dependent (bottom) pH_i recovery of CAFs cultured at conditions detailed in panel (C). All data follow normal distribution and are depicted as mean ± SEM, whereas data in (e) is represented as median ± 95% CI. Statistical tests in (D) and € were performed with two-tailed unpaired Student's t-tests.

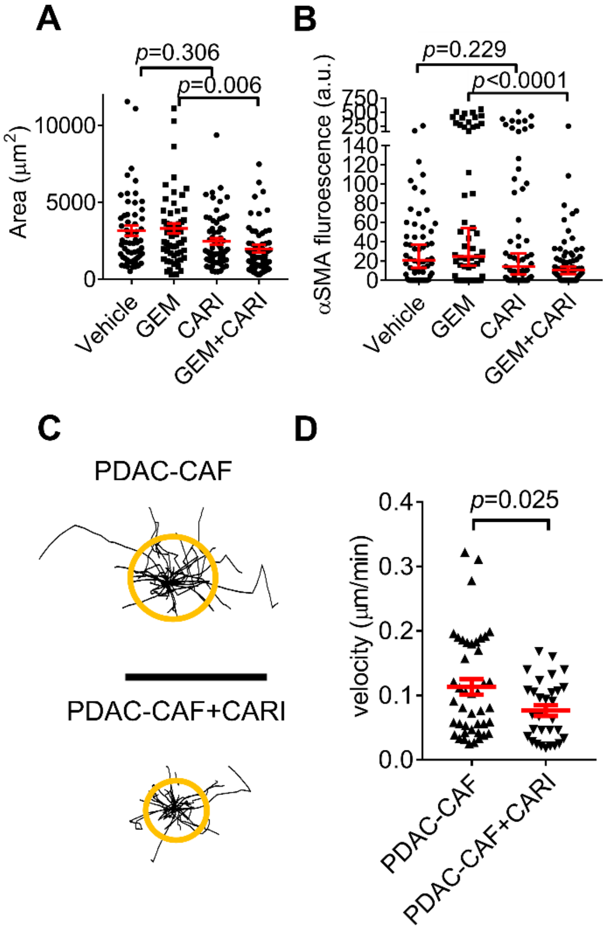

Supplementary Figure 5. Cariporide decreases PDAC-derived CAF area and migration velocity

A Scatter plot shows quantification of cell areas derived from immunocytochemistry depicted in Figure 6C-D. $n_{Vehicle}$=61, n_{GEM}=59, n_{CARI}=63, $n_{GEM+CARI}$=70 from N≥3 mice. **B** Scatter plot shows αSMA fluorescence intensity derived from Figure 6C-D (n/N see (A)) **C** Comparison of cell migration behavior of untreated (top) versus cariporide-treated (bottom) PDAC-bearing mouse-derived CAFs. Trajectories of individual CAFs are shown by individual black lines. Trajectories of the treatment groups are always normalized to common starting points. Orange circles highlight the mean displacement of cells in each population. Scale bar = 20 µm. **D** Mean cell migration velocities of individual CAFs were calculated from the trajectories in panel (a). Data points depict mean velocities of $n_{PDAC\text{-}PSC}$=43, $n_{PDAC\text{-}PSC+CARI}$ =30, cells from N=3 mice. All data follow normal distribution and are depicted as mean ± SEM. Statistical comparison in (A) and (B) were performed with one-way ANOVA with Tukey's post hoc test, whereas in (D) and with two-tailed unpaired Student's t-test.

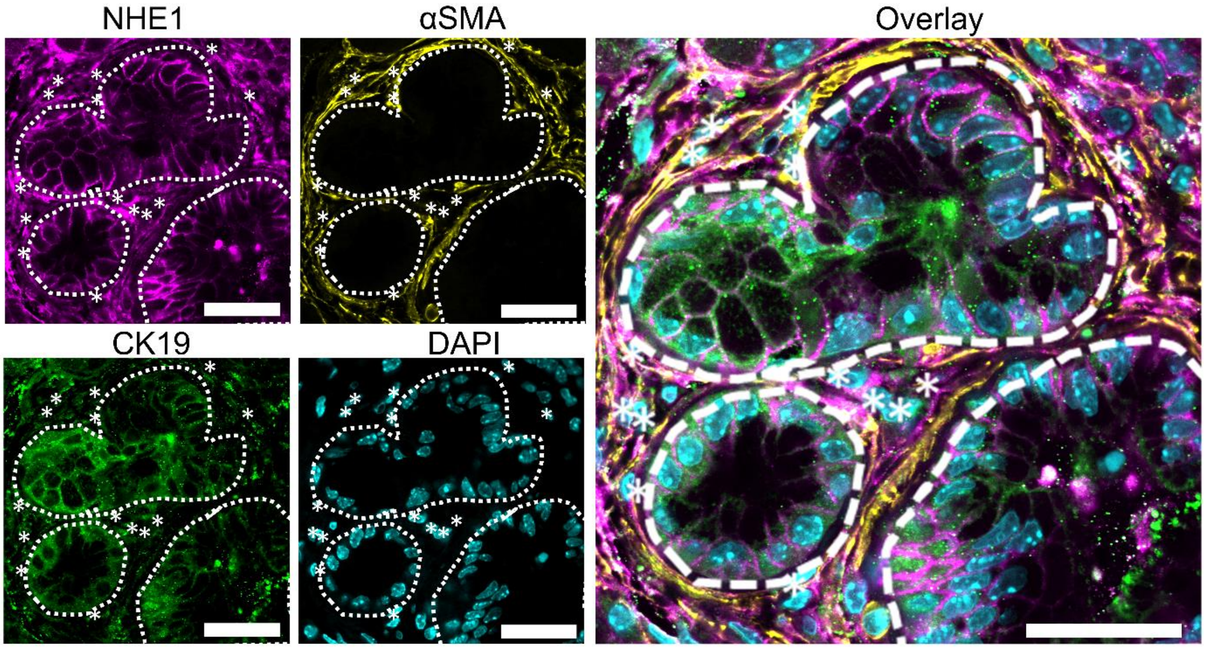

Supplementary Figure 6. NHE1 is ubiquitously expressed in PDAC

Representative immunohistochemistry image (from N=5) of NHE1 (magenta) present in the plasma membranes of most cells in PDAC: NHE1 is expressed, amongst others, in αSMA positive (yellow) CAFs (*) in CK19 positive (green) tumorous ducts (dotted line). Nuclei are marked by DAPI. Scale bar = 30 µm

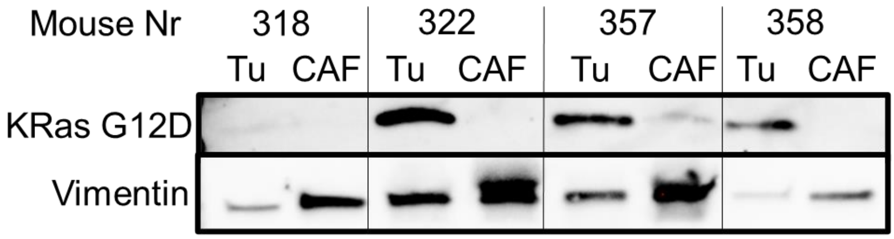

Supplementary Figure 7. KPfC-derived tumor cells but not CAFs carry the KRas G12D mutation

Western blot of the KRas G12D mutation (top lane) compared to vimentin (bottom lane) in tumor cells (Tu) and cancer-associated fibroblasts (CAF) from N=4 KPfC mice.

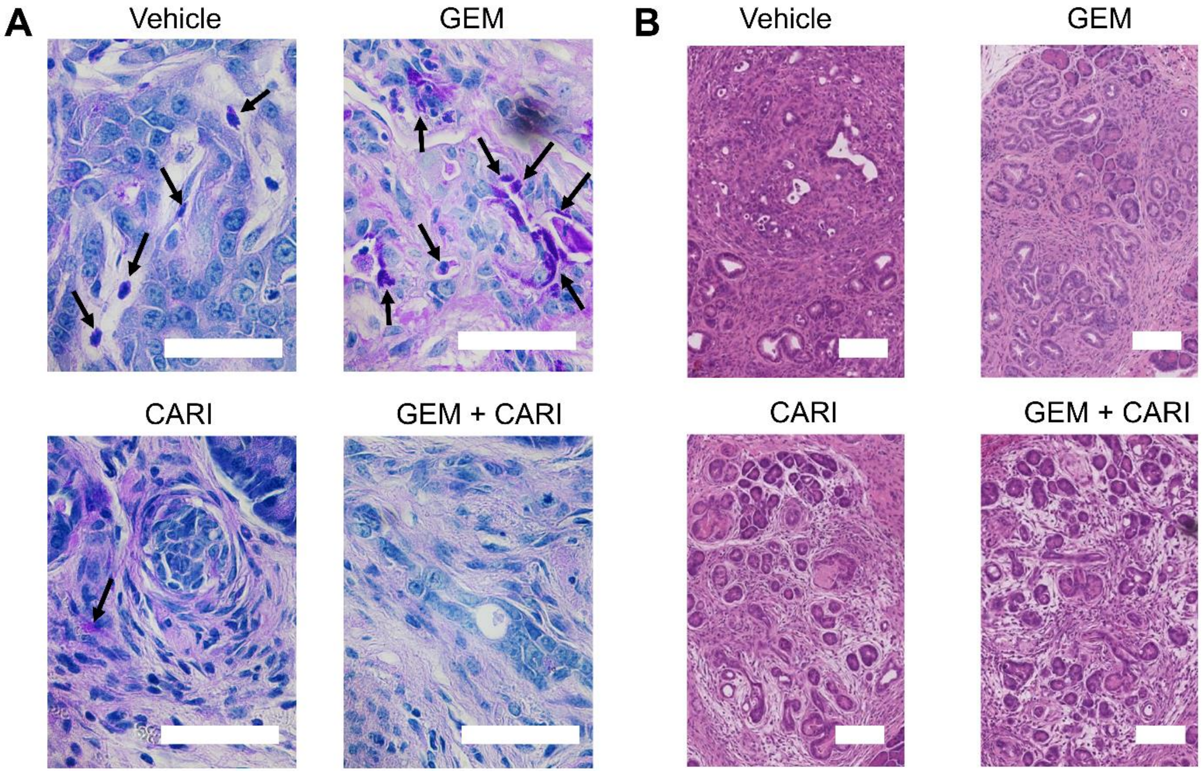

Supplementary Figure 8. Cariporide treatment alters immune infiltration in PDAC

A Representative Periodic acid-Schiff (PAS)-stained KPfC mouse tissue sections (from N≥10) after vehicle, gemcitabine (GEM), cariporide (CARI), and gemcitabine + cariporide (GEM+CARI) therapy highlights deposits of glycogen inside cells. Cells of innate immunity such as neutrophils (arrows) utilize glycogen and are thus PAS^{+} (purple), in contrast to e.g., lymphocytes. Scale bar = 50 µm. **B** Representative H+E images (from N≥10) demonstrating the altered tissue architecture of PDAC nodes after therapy.
